# Abnormal asymmetries in subcortical brain volume in early adolescents with subclinical psychotic experiences

**DOI:** 10.1038/s41398-018-0312-6

**Published:** 2018-11-28

**Authors:** Naohiro Okada, Noriaki Yahata, Daisuke Koshiyama, Kentaro Morita, Kingo Sawada, Sho Kanata, Shinya Fujikawa, Noriko Sugimoto, Rie Toriyama, Mio Masaoka, Shinsuke Koike, Tsuyoshi Araki, Yukiko Kano, Kaori Endo, Syudo Yamasaki, Shuntaro Ando, Atsushi Nishida, Mariko Hiraiwa-Hasegawa, Kiyoto Kasai

**Affiliations:** 1 Department of Neuropsychiatry, Graduate School of Medicine, The University of Tokyo, Tokyo, Japan; 2 International Research Center for Neurointelligence (WPI-IRCN), The University of Tokyo Institutes for Advanced Study (UTIAS), The University of Tokyo, Tokyo, Japan; 3 Department of Molecular Imaging and Theranostics, National Institute of Radiological Sciences, National Institutes for Quantum and Radiological Science and Technology, Chiba, Japan; 4 Department of Psychiatry, Teikyo University School of Medicine, Tokyo, Japan; 5 UTokyo Institute for Diversity and Adaptation of Human Mind (UTIDAHM), The University of Tokyo, Tokyo, Japan; 6 Department of Child Psychiatry, Graduate School of Medicine, The University of Tokyo, Tokyo, Japan; 7 Department of Psychiatry and Behavioral Sciences, Tokyo Metropolitan Institute of Medical Science, Tokyo, Japan; 8 Department of Evolutionary Studies of Biosystems, School of Advanced Sciences, The Graduate University for Advanced Studies (SOKENDAI), Kanagawa, Japan

## Abstract

Subcortical structures may have an important role in the pathophysiology of psychosis. Our recent mega-analysis of structural magnetic resonance imaging (MRI) data has reported subcortical volumetric and lateralization alterations in chronic schizophrenia, including leftward asymmetric increases in pallidal volume. The question remains, however, whether these characteristics may represent vulnerability to the development of psychosis or whether they are epiphenomena caused by exposure to medication or illness chronicity. Subclinical psychotic experiences (SPEs) occur in some adolescents in the general population and increase the odds of developing psychosis in young adulthood. Investigations into the association between SPEs and MRI-measured volumes of subcortical structures in the general adolescent population would clarify the issue. Here, we collected structural MRI data in a subsample (10.5–13.3 years old) of a large-scale population-based cohort and explored subcortical volume and lateralization alterations related to SPEs (*N* = 203). Adolescents with SPEs demonstrated significant volumetric increases in the left hippocampus, right caudate, and right lateral ventricle, as well as a marginally significant increase in the left pallidum. Furthermore, adolescents with SPEs showed significantly more leftward laterality of pallidal volume than individuals without SPEs, which replicates our mega-analysis findings in chronic schizophrenia. We suggest that leftward asymmetries in pallidal volume already present in early adolescence may underlie the premorbid predisposition for developing psychosis in later life.

## Introduction

Subcortical structures, including the basal ganglia as well as parts of the limbic system, are not only important players in learning^1^, emotion^2^, motor control^3^, and attention^4^ but also integrally involved in higher-order executive functions such as working memory^5^ and inhibitory control^6^. These psychological functions are often impaired in patients with schizophrenia or psychosis, and therefore it is beneficial to focus on characteristics of subcortical regions in these patients. We recently reported subcortical volumetric and lateralization alterations in schizophrenia, including leftward asymmetric increases in pallidal volume, through a large-scale mega-analysis of structural magnetic resonance imaging (MRI) data in a study from the Cognitive Genetics Collaborative Research Organization (COCORO) consortium^7^. In a secondary analysis study, we showed that daily antipsychotics dosage, but not the duration of illness or type of antipsychotic medication, may at least partially explain both the left pallidal volume increase and leftward pallidal volumetric laterality in schizophrenia^8^. The question remains, however, whether these characteristics may represent vulnerability to the development of psychosis or whether they are epiphenomena caused by exposure to medication or illness chronicity.

Subclinical psychotic (delusional and hallucinatory) experiences (SPEs), often called psychotic-like experiences, occur in individuals in the general population as a nonpathological subthreshold phenotype^9^, which suggests an underlying continuum of psychosis from subclinical to clinical levels^10^. According to longitudinal birth cohort studies, SPEs in early adolescence are a risk factor for the later onset of schizophrenia or schizophreniform disorder^11,12^. While a previous meta-analysis revealed a 5% prevalence rate of SPEs in the general population^13^, the rate of SPEs may be much higher among early adolescents^14^.

The underlying bases of SPEs have recently been examined. Twin studies reveal larger concordance of SPEs between monozygotic twins than between dizygotic twins, suggesting the heritability of SPEs^15^. A family cohort study reveals a positive relationship between mothers’ psychosis-spectrum disorders and children’s SPEs^16^. It is thus assumed that genetic factors may have an influence on SPEs. However, the genetic factors influencing SPEs are not yet specified^17,18^. Neural substrates of SPEs have recently been explored in some neuroimaging studies using structural MRI^19–21^, diffusion-tensor imaging (DTI)^20,22,23^, task-related functional MRI (fMRI)^20,24,25^, and resting-state fMRI (rsfMRI)^26^. We hypothesized that the results of the previous findings on volumetric alterations in schizophrenia^7,27^ would be replicated in adolescents with SPEs for each subcortical region. To our knowledge, however, no previous study has focused on the volume of subcortical structures in a large-scale sample from the general population.

The brain dynamically develops throughout childhood to adolescence. During adolescence, extensive synaptic pruning and myelination occur in the brain^28^. These processes are associated with cortical thinning^29^ and white matter volume increases^30^, that can be observed using MRI techniques. Such brain maturation in adolescence can be affected by sex hormone changes and secondary sexual characteristics during puberty^31^. Recent studies reported that subcortical structures can be volumetrically affected by pubertal development^32–34^. Thus, specifically when examining the association between subcortical volumes and SPEs in adolescence, it would be ideal to regress out the effects of the pubertal stage or to limit the scope of analysis to prepubertal adolescents.

Here, given this context, we collected structural MRI data in a subsample (10.5–13.3 years old) of a large-scale population-based cohort. First, we investigated subcortical volumetric and lateralization alterations in adolescents with SPEs. Second, we examined them in detail, with special attention paid to the globus pallidus, which was shown to be important in our previous study of chronic schizophrenia. Potential confounding factors, including pubertal stage, were considered. The advantage of our study design is that a number of participants were recruited from an ongoing population-based cohort study involving 3171 early adolescents (Tokyo TEEN Cohort: TTC, http://ttcp.umin.jp/). This sort of study has recently been called “population neuroscience”^35^ and can minimize selection biases that are sometimes seen in laboratory-based neuroimaging studies. We expected the current study to overcome the limitations of our previous studies and to elucidate the detailed mechanisms of subcortical volumetric alterations in schizophrenia.

## Materials and methods

### Participants and ethics

This study was conducted as part of the population neuroscience study of the TTC (pn-TTC) study, in which 301 early adolescents were recruited from the general population. All study participants and their primary parents provided written informed consent, and the appropriate ethics review boards approved the study design. The detailed methods of participant recruitment and ethics are described in Supplementary Method 1.

### Scanning and image processing

Each subject underwent T1-weighted MRI. Imaging data were processed with FreeSurfer software version 5.3^36^. The details of this procedure are provided in Supplementary Method 2.

### Psychological and physical evaluation

The 7-item Adolescent Psychotic-like Symptom Screener (APSS)^37^ was used to screen for SPEs. In addition, Tanner stages^38^ were used to assess pubertal development, since the effect of pubertal development on subcortical brain volumes should be non-negligible^32–34^. The details of these scales are presented in Supplementary Method 3.

### Subject selection

A total of 203 adolescents were included in this study. The details of the selection procedure are described in Supplementary Method 4. The basic demographic data are described in Table 1. A histogram of APSS scores is shown in Supplementary Figure 1.

**Table 1:** A cross-table of Tanner stage, subclinical psychotic experiences (SPEs), and sex for the participants included in this study

Sex			Tanner stage						Total
			1	2	3	4	5	Data missing	
Boys	SPE	Negative	17	32	16	6	0	1	72
		Positive	5	14	10	5	0	0	34
	Total		22	46	26	11	0	1	106
Girls	SPE	Negative	17	20	17	11	0	2	67
		Positive	7	7	9	7	0	0	30
	Total		24	27	26	18	0	2	97
Total	SPE	Negative	34	52	33	17	0	3	139
		Positive	12	21	19	12	0	0	64
	Total		46	73	52	29	0	3	203

*SPE* subclinical psychotic experience

### Statistical analysis

#### Overall statistical strategy

All statistical analyses were conducted using SPSS version 19.0.0 or 24.0.0 (IBM, New York, NY, USA). Since we hypothesized and examined whether the results of the previous studies on schizophrenia^7,27^ would be replicated in adolescents with SPEs for each subcortical region, we set the type I error rate (*p* value) at 0.05.

Basic demographic data are depicted in Table 1. Prior to the main analysis, we investigated sex and age differences between the SPE-negative [SPE(−)] and SPE-positive [SPE(+)] group by using *χ*^2^ test and *t*-test, respectively. Additionally, we examined sex and age differences in subcortical regional volumes in all participants by using *t*-test and Spearman’s rank test, respectively. Furthermore, we examined associations between subcortical regional volumes and Tanner stages by using Spearman’s rank test.

#### Subcortical volume analysis

##### Alterations of subcortical regional volumes in adolescents with SPEs

First, the means and standard deviations of subcortical regional volumes were calculated in the SPE(−) and SPE(+) groups. Second, we explored group differences in subcortical regional volumes. First, we investigated group differences by using general linear models controlling for the effects of age, sex, and intracranial volume (ICV) (Model 1, main model), which might be potential confounding factors, as employed in the previous studies^7,27^. Then, we used general linear models without covariates (Model 2) and models controlling for age (Model 3) and for age and sex (Model 4). Moreover, as developmental levels may affect subcortical regional volumes, we employed general linear models controlling for the effect of age, sex, ICV, and Tanner stage (Model 5). Cohen’s *d* effect sizes were calculated as the ratio of group difference to the pooled standard deviation.

##### Intra-group subcortical volume laterality in adolescents without and with SPEs

To assess laterality for subcortical volumes, we employed a laterality index (LI), defined as the ratio [(left − right)/(left + right)]; this is commonly used to evaluate brain structural asymmetry. LIs can range from −1 to 1 and a positive LI means a leftward asymmetry.

First, the means and standard deviations of LIs of subcortical regional volumes were calculated in the SPE(−) and SPE(+) groups. Second, one-sample *t*-tests were conducted to evaluate whether mean LIs were significantly different from zero. For each group, Cohen’s *d* effect sizes for LIs were calculated as the ratio of the mean (i.e., mean difference to zero) to standard deviation.

##### Group differences in subcortical volume laterality between adolescents without and with SPEs

We examined the differences in LIs of subcortical regional volumes between the SPE(−) and SPE(+) groups. First, we calculated group differences by using general linear models controlling for the effect of age and sex (Model 1, main model), as employed in our previous study^7^. Then, we used general linear models without covariates (Model 2) and models controlling for age (Model 3) and for age, sex, and Tanner stage (Model 4). Cohen’s *d* effect sizes were calculated as the ratio of group difference to the pooled standard deviation.

#### Detailed analysis on the globus pallidus

##### Alterations of pallidal volume and its laterality in adolescents with SPEs within each sex

As sex may affect pallidal volume and its laterality, we investigated group differences in them for each sex group.

First, we investigated group differences in left and right pallidal volume by using general linear models controlling for the effect of age and ICV (Model 1, main model). Then, we employed general linear models without covariates (Model 2) and models controlling for age (Model 3) and for age, ICV, and Tanner stage (Model 4).

Second, we investigated group differences in LIs of pallidal volume by using general linear models controlling for the effect of age (Model 1, main model). Then, we used general linear models without covariates (Model 2) and models controlling for the effect of age and Tanner stage (Model 3).

##### Comparisons of pallidal volume and its laterality between the SPE(−) and SPE(+) groups exclusively in adolescents in Tanner stage I

As sexual maturation levels may affect pallidal volume and its laterality, we excluded participants whose Tanner stages were ≥2 in this sub-analysis.

First, we explored group differences in left and right pallidal volume by using general linear models controlling for the effect of age, sex, and ICV (Model 1, main model). Then, we used general linear models without covariates (Model 2) and models controlling for age (Model 3) and for age and sex (Model 4).

Second, we examined group differences in LIs of pallidal volume by using general linear models controlling for the effect of age and sex (Model 1, main model). Then, we used general linear models without covariates (Model 2) and models controlling for the effect of age (Model 3).

We also conducted separate analyses for each sex. We investigated group differences in left and right pallidal volume by using general linear models controlling for the effect of age and ICV (Model 1, main model). Then, we used general linear models without covariates (Model 2) and models controlling for the effect of age (Model 3).

Moreover, we performed separate analyses for each sex to investigate group differences in LIs of pallidal volume by using general linear models controlling for the effect of age in the main analysis (Model 1, main model). Then, we used general linear models without covariates (Model 2).

##### Hemispheric differences in pallidal volume

We examined hemispheric differences in pallidal volume. First, for all participants, we performed a two-way repeated-measures analysis of covariance (ANCOVA) using group [SPE(−)/SPE(+)] and sex (male/female) as between-subject factors, hemisphere (left/right) as a within-subject factor, and age and ICV as covariates. Second, as sexual maturation levels may have some influences, we conducted the same analysis exclusively for adolescents in Tanner stage 1.

##### Correlation analysis for the whole group

We examined associations of psychotic-like symptoms with pallidal volume and its laterality.

First, we calculated beta coefficients of APSS total score to left and right pallidal volume by using general linear models controlling for the effect of age, sex, and ICV (Model 1, main model). Then, we used general linear models without covariates (Model 2) and controlling for the effect of age (Model 3), age and sex (Model 4), and age, sex, ICV, and Tanner stage (Model 5).

Second, we calculated beta coefficients of APSS total score to LIs of pallidal volume by using general linear models controlling for the effect of age and sex (Model 1, main model). Then, we used general linear models without covariates (Model 2) and controlling for the effect of age (Model 3), and age, sex, and Tanner stage (Model 4).

##### Correlation analysis exclusively for adolescents in Tanner stage I

As sexual maturation levels may have some influences, we conducted the same analysis exclusively for adolescents in Tanner stage 1.

First, we calculated beta coefficients of APSS total score to left and right pallidal volume by using general linear models controlling for the effect of age, sex, and ICV (Model 1, main model). Then, we used general linear models without covariates (Model 2) and models controlling for age (Model 3) and for age and sex (Model 4).

Second, we calculated beta coefficients of APSS total score to LIs of pallidal volume by using general linear models controlling for the effect of age and sex (Model 1, main model). Then, we used general linear models without covariates (Model 2) and models controlling for the effect of age (Model 3).

#### Power analysis

We conducted power analyses to ensure that the current study had statistically sufficient power. A priori power analyses (*α* = 0.05, 1 − *β* = 0.80, two-sided tests) using G*Power 3.1.9.2.^39^ revealed that the medium effect sizes for a two-sample *t*-test (*d* = 0.50), those for a correlational analysis (*r* = 0.30), those for a multiple regression analysis (*f*^2^ = 0.15), and those for a 2 × 2 *χ*^2^ test (*w* = 0.30)^40^ could be detected in a total sample size of 128 (64 for each group), 82, 55, and 88, respectively. The sample sizes in all main analyses in this study were adequate.

## Results

### Overall sex and age effects

We investigated sex and age differences between the SPE(−) and SPE(+) groups. We found no significant group differences in sex (*p* = 0.86) or age (*p* = 0.52) (Supplementary Table 1). Moreover, we examined sex and age differences in subcortical regional volumes in all participants. We found significantly larger regional volumes in boys than in girls for all subcortical regions except the lateral ventricles and bilateral caudate (*p* = 3.3 × 10^−12^–2.8 × 10^−2^), in which we found no sex differences (Supplementary Table 2). Age had significant negative correlations with right putamen (*p* = 4.5 × 10^−2^) and right accumbens volume (*p* = 7.3 × 10^−3^), while no age correlations with the other subcortical regions were found (Supplementary Table 3). Furthermore, we examined associations between subcortical regional volumes and Tanner stages. We found significant negative correlations between Tanner stages and left putamen (*p* = 4.3 × 10^−2^), right putamen (*p* = 7.4 × 10^−4^), and left accumbens volume (*p* = 1.3 × 10^−3^), while there were no correlations of Tanner stages with the other subcortical volumes (Supplementary Table 4).

### Alterations of subcortical regional volumes in adolescents with SPEs

**Fig. 1:**
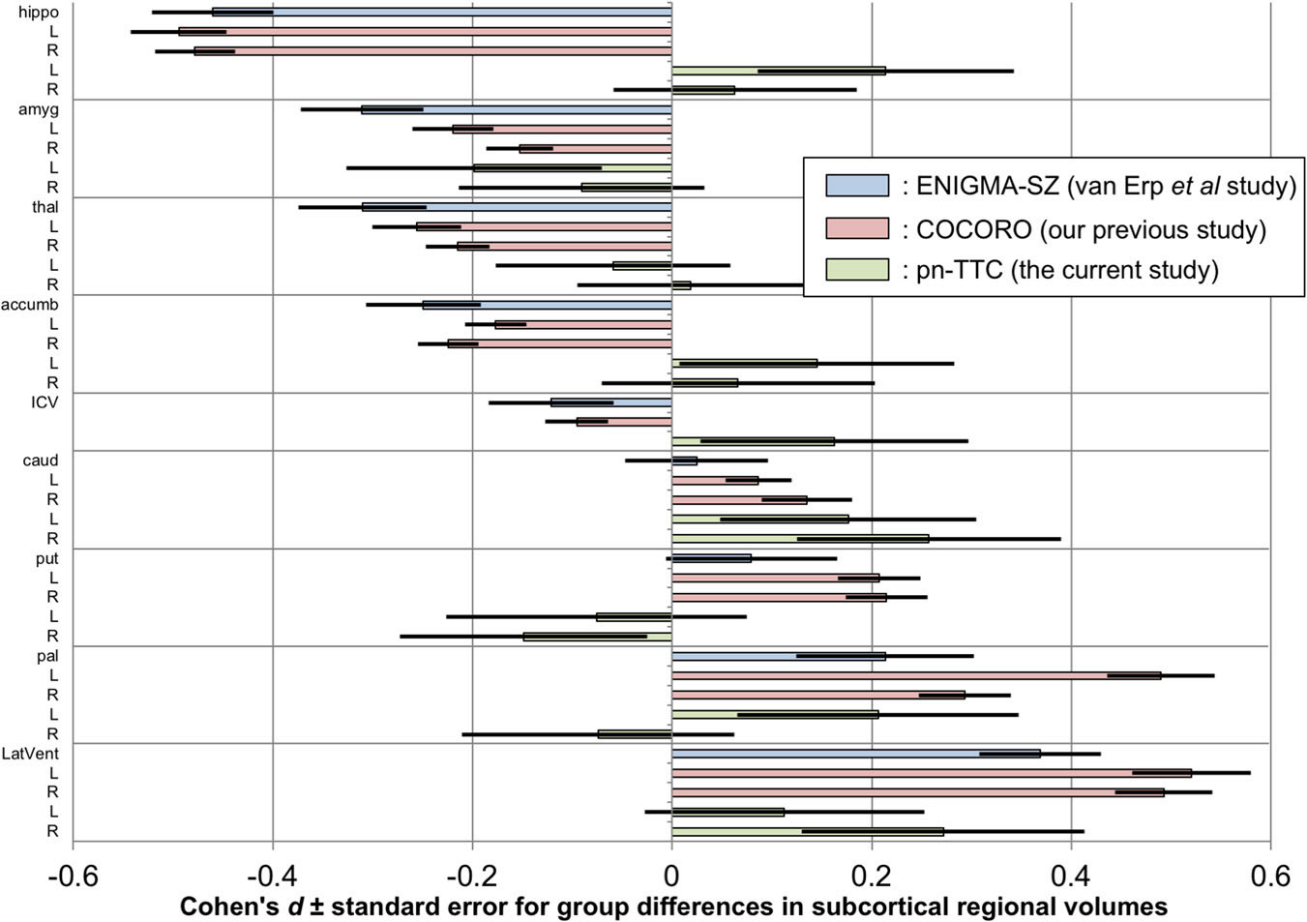
Cohen’s *d* effect sizes ± standard errors for differences in subcortical regional volumes between the group with subclinical psychotic experiences (SPEs) and the group without SPEs. The results of the previous study from the Enhancing Neuroimaging Genetics through Meta-analysis (ENIGMA) Schizophrenia (ENIGMA-SZ) working group (schizophrenia–control contrast), those of our previous study from the Cognitive Genetics Collaborative Research Organization (COCORO) consortium (schizophrenia–control contrast), and those of the current work from the population-neuroscience study of the Tokyo TEEN Cohort (pn-TTC) project (SPE-positive–SPE-negative contrast) are merged. Between-group differences in subcortical regional volumes adjusted for age, sex, and intracranial volume (ICV) were obtained. An overall effect size was calculated as the ratio of the overall difference to the pooled standard deviation. A positive effect size means that individuals with SPEs and those with schizophrenia had larger volumes than those without. *Abbreviations*: ENIGMA, Enhancing Neuroimaging Genetics through Meta-analysis; ENIGMA-SZ, ENIGMA Schizophrenia; COCORO, Cognitive Genetics Collaborative Research Organization; pn-TTC, population-neuroscience study of the Tokyo TEEN Cohort; Lhippo, left hippocampus; Rhippo, right hippocampus; Lamyg, left amygdala; Ramyg, right amygdala; Lthal, left thalamus; Rthal, right thalamus; Laccumb, left accumbens; Raccumb, right accumbens; Lcaud, left caudate; Rcaud, right caudate; Lput, left putamen; Rput, right putamen; Lpal, left pallidum; Rpal, right pallidum; LLatVent, left lateral ventricle; RLatVent, right lateral ventricle

The means and standard deviations of subcortical regional volumes were calculated in the SPE(−) and SPE(+) groups (Supplementary Table 5). We then explored group differences in subcortical regional volumes controlling for the effect of age, sex, and ICV (Model 1, main model); no covariates (Model 2); age (Model 3); age and sex (Model 4); and age, sex, ICV, and Tanner stage (Model 5) (Supplementary Tables 6–10). We found significantly larger volumes of the left hippocampus (*p* = 0.043–0.044, Models 2 and 4), right caudate (*p* = 0.022–0.023, Models 2–4), and right lateral ventricle volume (*p* = 0.026–0.026, Models 2–4) in the SPE(+) group than in the SPE(−) group. For the globus pallidus, the left-side volume was larger in the SPE(+) group than in the SPE(−) group at a marginal significance level (*p* = 0.078–0.080, Models 2–4), although there was no significant group difference in the right-side volume. The Cohen’s *d* effect sizes and standard errors for group differences in subcortical regional volumes (Model 1) are depicted in Fig. 1, where the results of the previous study from the Enhancing Neuroimaging Genetics through Meta-analysis (ENIGMA) Schizophrenia (ENIGMA-SZ) working group^27^, those of our previous study from COCORO^7^, and those of the current study from the pn-TTC project are merged.

### Intra-group subcortical volume laterality in adolescents without and with SPEs

**Fig. 2:**
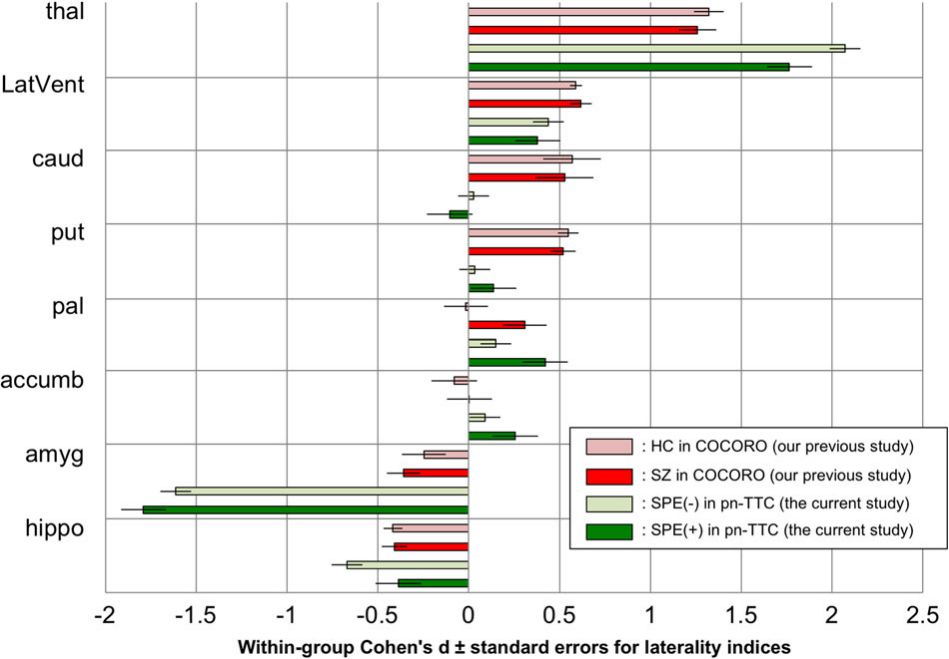
Within-group Cohen’s *d* effect sizes ± standard errors for laterality indices (LIs) of subcortical regional volumes in the subclinical psychotic experience (SPE)-negative [SPE(−)] and SPE-positive [SPE(+)] groups. The results of our previous study from the Cognitive Genetics Collaborative Research Organization (COCORO) consortium, which included patients with schizophrenia and healthy controls, and those of the current study from the population-neuroscience study of the Tokyo TEEN Cohort (pn-TTC) project are merged. An LI was defined as the ratio [(left − right)/(left + right)]. An overall effect size for LIs was calculated as the ratio of the overall mean LIs to the overall standard deviation. A positive effect size demonstrates a leftward asymmetry. *Abbreviations*: COCORO, Cognitive Genetics Collaborative Research Organization; pn-TTC, population-neuroscience study of the Tokyo TEEN Cohort; SPE, subclinical psychotic experience; thal, thalamus; LatVent, lateral ventricle; caud, caudate; put, putamen; pal, pallidum; accumb, accumbens; amyg, amygdala; hippo, hippocampus

The means and standard deviations of LIs of subcortical regional volumes were calculated in the SPE(−) and SPE(+) groups (Supplementary Table 11). Next, we evaluated whether mean LIs were significantly different from zero (Supplementary Table 11). In the SPE(−) group, the LIs of the lateral ventricle (*p* = 7.6 × 10^−7^) and thalamus (*p* = 6.4 × 10^−52^) were significantly positive and those of hippocampus (*p* = 8.2 × 10^−13^) and amygdala (*p* = 2.2 × 10^−40^) were significantly negative, while those of the caudate, putamen, and accumbens were not significantly different from zero and those of the pallidum were positive with marginal significance (*p* = 0.08). In the SPE(+) group, the LIs of the lateral ventricle (*p* = 3.4 × 10^−3^), thalamus (*p* = 3.5 × 10^−21^), pallidum (*p* = 1.3 × 10^−3^), and accumbens (*p* = 4.5 × 10^−2^) were significantly positive and those of the hippocampus (*p* = 2.9 × 10^−3^) and amygdala (*p* = 1.8 × 10^−21^) were significantly negative, while those of the caudate and putamen were not significantly different from zero. The Cohen’s *d* effect sizes and standard errors for the LIs of subcortical regional volumes in each group are depicted in Fig. 2, where the results of our previous study from COCORO^7^ and those of the current study from the pn-TTC project are merged.

### Group differences in subcortical volume laterality between adolescents without and with SPEs

**Fig. 3:**
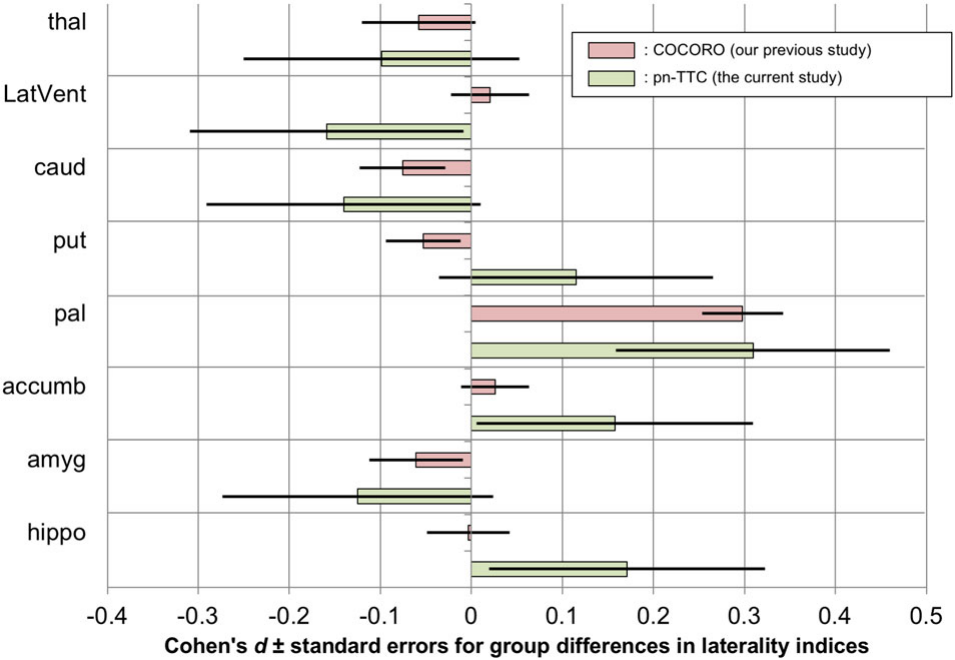
Cohen’s *d* effect sizes ± standard errors for differences in laterality indices (LIs) of subcortical regional volumes between the group with subclinical psychotic experiences (SPEs) and the group without SPEs. The results of our previous study from the Cognitive Genetics Collaborative Research Organization (COCORO) consortium (schizophrenia–control contrast) and those of the current study from the population-neuroscience study of the Tokyo TEEN Cohort (pn-TTC) project are merged. An LI was defined as the ratio [(left − right)/(left + right)]. Between-group differences in LIs adjusted for covariates such as age and sex were obtained. An overall effect size was calculated as the ratio of the overall difference to the pooled standard deviation. A positive effect size means that individuals with SPEs and those with schizophrenia showed larger LI than those without. *Abbreviations*: COCORO, Cognitive Genetics Collaborative Research Organization; pn-TTC, population-neuroscience study of the Tokyo TEEN Cohort; thal, thalamus; LatVent, lateral ventricle; caud, caudate; put, putamen; pal, pallidum; accumb, accumbens; amyg, amygdala; hippo, hippocampus

We examined the differences in the LIs of subcortical regional volumes between the SPE(−) and SPE(+) groups controlling for the effect of age and sex (Model 1, main model); no covariates (Model 2); age (Model 3); and age, sex, and Tanner stage (Model 4) (Supplementary Tables 12–15). We found a significantly larger LI of pallidal volume in the SPE(+) group than in the SPE(−) group (*p* = 0.035–0.043, Models 1–4). The Cohen’s *d* effect sizes and standard errors for LIs of subcortical regional volumes in each group (Model 1) are depicted in Fig. 3, where the results of our previous study from COCORO^7^ and those of the current study from the pn-TTC project are merged.

### Detailed analysis of the globus pallidus

**Fig. 4:**
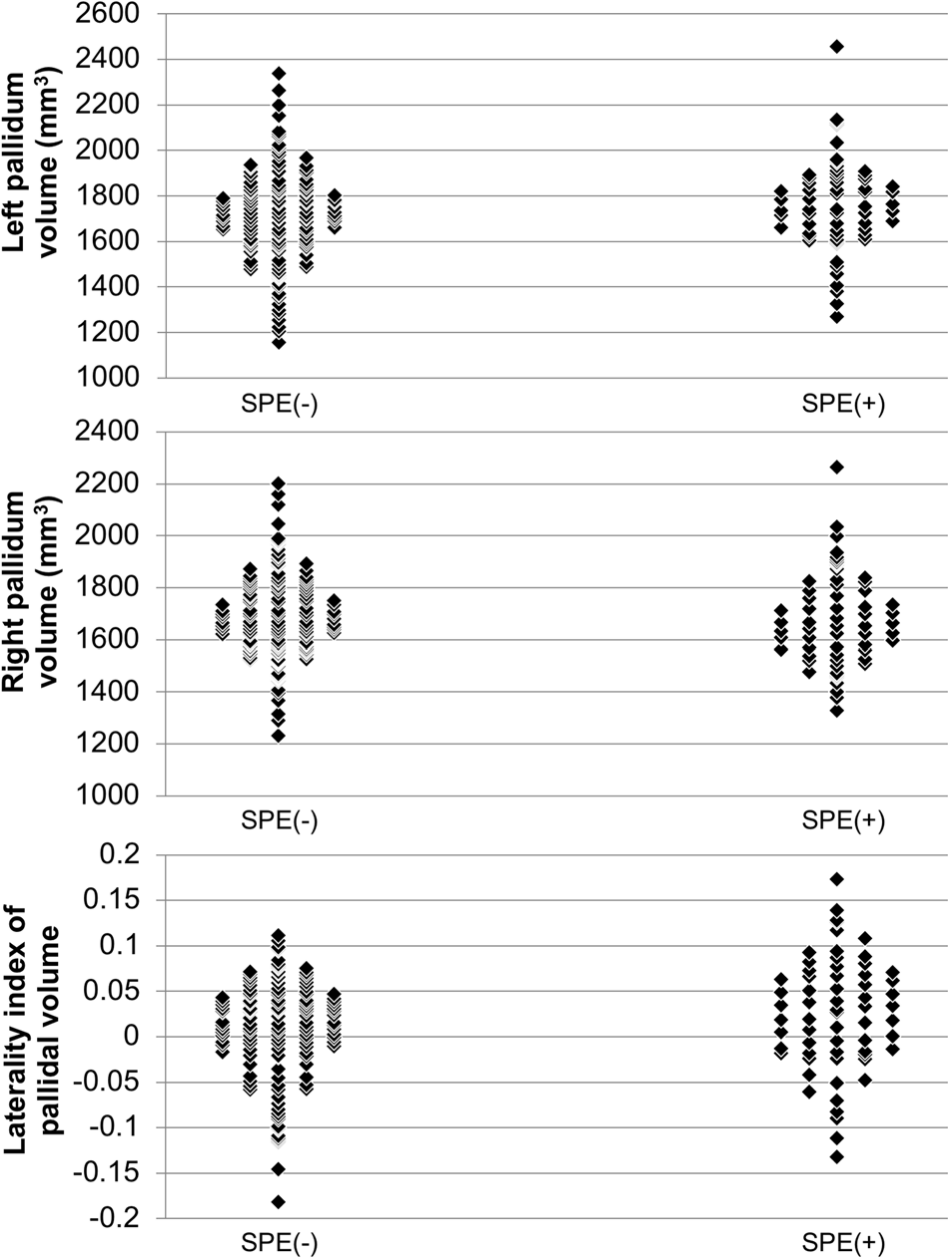
Distribution plots for left and right pallidal volume and laterality indices (LIs) of pallidal volume in the subclinical psychotic experience (SPE)-negative [SPE(−)] and SPE-positive [SPE(+)] groups. An LI was defined as the ratio [(left − right)/(left + right)]. A positive LI demonstrates a leftward asymmetry. *Abbreviation*: SPE, subclinical psychotic experience

Prior to detailed analyses of the globus pallidus, we here show distribution plots of left and right pallidal volume, as well as LIs of pallidal volume, in the SPE(−) and SPE(+) groups (Fig. 4).

### Alterations of pallidal volume and its laterality in adolescents with SPEs within each sex

We investigated group differences in left and right pallidal volume in each sex controlling for the effect of age and ICV (Model 1, main model); no covariates (Model 2); age (Model 3); and age, ICV, and Tanner stage (Model 4) (Supplementary Table 16). Left pallidal volume was larger to a marginally significant degree in SPE(+) boys than in SPE(−) boys (*p* = 0.071–0.076, Models 1 and 4). There were no significant differences in pallidal volume between SPE(+) and SPE(−) girls.

We then investigated group differences in LIs of pallidal volume in each sex controlling for the effect of age (Model 1, main model); no covariates (Model 2); and age and Tanner stage (Model 3) (Supplementary Table 17). LIs of pallidal volume were larger to a marginally significant degree in SPE(+) boys than in SPE(−) boys (*p* = 0.075–0.087, Models 1 and 2). There were no significant differences in pallidal volume between SPE(+) and SPE(−) girls.

### Comparisons of pallidal volume and its laterality between the SPE(−) and SPE(+) groups exclusively in adolescents in Tanner stage I

We explored group differences in left and right pallidal volume controlling for the effect of age, sex, and ICV (Model 1, main model); no covariates (Model 2); age (Model 3); and age and sex (Model 4) (Supplementary Table 18). The SPE(+) group showed significantly larger left pallidal volume than the SPE(−) group (*p* = 0.011–0.035, Models 1–4), while there were no significant group differences in right pallidal volume.

We then examined group differences in LIs of pallidal volume controlling for the effect of age and sex (Model 1, main model); no covariates (Model 2); and age (Model 3) (Supplementary Table 19). The SPE(+) group demonstrated a significantly higher LI of pallidal volume than the SPE(−) group (*p* = 7.5 × 10^−4^–1.3 × 10^−3^, Models 1–3).

We also conducted separate analyses for each sex. We investigated group differences in left and right pallidal volume in each sex controlling for the effect of age and ICV (Model 1, main model); no covariates (Model 2); and age (Model 3) (Supplementary Table 20). In boys, the SPE(+) group showed larger left pallidal volume to a marginally significant degree than the SPE(−) group (*p* = 0.052–0.078, Models 1 and 3). In girls, the SPE(+) group showed significantly larger left pallidal volume (*p* = 0.044, Model 2) than the SPE(−) group.

Moreover, we investigated group differences in LIs of pallidal volume controlling for the effects of age (Model 1, main model) and no covariates (Model 2) in each sex group (Supplementary Table 21). In both sex groups, the SPE(+) group demonstrated higher LI of pallidal volume than the SPE(−) group, marginally in boys and significantly in girls (*p* = 0.058–0.064, Models 1 and 2 in boys; *p* = 0.013–0.025, Models 1 and 2 in girls).

### Hemispheric differences in pallidal volume

For all participants, we performed a two-way repeated measures ANCOVA using the group [SPE(+)/SPE(−)] and sex (male/female) as between-subject factors, hemisphere (left/right) as a within-subject factor, and age and ICV as covariates. We found a marginally significant effect of hemisphere-by-group interaction (*p* = 0.057), while there was no significant effect of hemisphere (*p* = 0.56), hemisphere by age (*p* = 0.46), hemisphere by ICV (*p* = 0.71), hemisphere by sex (*p* = 0.85), or hemisphere by sex by group (*p* = 0.60). We then conducted the same analysis exclusively for adolescents in Tanner stage 1. We found a significant effect of hemisphere-by-group interaction (*p* = 1.3 × 10^−3^), while there was no significant effect of hemisphere (*p* = 0.27), hemisphere by age (*p* = 0.16), hemisphere by ICV (*p* = 0.49), hemisphere by sex (*p* = 0.58), or hemisphere by sex by group (*p* = 0.68).

### Correlation analysis for the whole group

We calculated beta coefficients of APSS total score to left and right pallidal volume controlling for the effect of age, sex, and ICV (Model 1, main model); no covariates (Model 2); age (Model 3); age and sex (Model 4); and age, sex, ICV, and Tanner stage (Model 5) (Supplementary Table 22). There was no significant correlation between APSS total score and left or right pallidal volume.

We then calculated beta coefficients of APSS total score to LIs of pallidal volume controlling for the effect of age and sex (Model 1, main model); no covariates (Model 2); age (Model 3); and age, sex, and Tanner stage (Model 4) (Supplementary Table 23). There was no significant correlation of APSS total score to LIs of pallidal volume.

### Correlation analysis exclusively for adolescents in Tanner stage I

We conducted the same analysis exclusively for adolescents in Tanner stage 1. We calculated beta coefficients of APSS total score to left and right pallidal volume controlling for the effect of age, sex, and ICV (Model 1, main model); no covariates (Model 2); age (Model 3); and age and sex (Model 4) (Supplementary Table 24). We found a significant positive correlation of APSS total score to left pallidal volume (*p* = 0.025–0.041, Models 1–4) but no significant correlation to right pallidal volume.

We then calculated beta coefficients of APSS total score to LIs of pallidal volume controlling for the effects of age and sex (Model 1, main model); no covariates (Model 2); and age (Model 3) (Supplementary Table 25). We found a significant positive correlation between APSS total score and LIs of pallidal volume (*p* = 0.011–0.016, Models 1–3).

## Discussion

In the current study, we collected structural MRI data in a subsample (10.5–13.3 years old) of a large-scale population-based cohort and explored subcortical volumetric and lateralization alterations in adolescents with SPEs. First, adolescents with SPEs demonstrated significant increases in left hippocampus, right caudate, and right lateral ventricle volume (Fig. 1). Second, both SPE-negative and SPE-positive adolescents commonly had leftward asymmetries of the lateral ventricle and thalamus, rightward asymmetries of the hippocampus and amygdala, and no asymmetries of the caudate or putamen, while the pallidum and accumbens showed no asymmetries in individuals without SPEs but leftward asymmetries in those with SPEs (Fig. 2). Third, laterality for pallidal volume was significantly more leftward in adolescents with SPEs than in those without (Fig. 3). Moreover, in additional analyses, we demonstrated marginally significantly larger volume in the left pallidum and more leftward asymmetry for pallidal volume in boys with SPEs than those without. Furthermore, by limiting the scope to Tanner stage 1, we revealed a larger volume in left pallidum and more leftward asymmetry for pallidal volume in adolescents with SPEs than those without.

Adolescents with SPEs demonstrated significant increases in left hippocampus, right caudate, and right lateral ventricle volume. Enlarged volumes of the right caudate and right ventricle in adolescents with SPEs are concordant with previous large-scale studies on chronic schizophrenia (Fig. 1)^7^, although caudate head volumes are decreased in first-episode psychosis^41^ and caudate volumes are normal in high-risk individuals^42^. It is unclear why right caudate volumes are increased in individuals with SPEs, but this may be an incipient alteration in potentially risky adolescents. Hippocampal volumes are decreased bilaterally in chronic schizophrenia^7,27,43^, in first-episode schizophrenia^44–46^, and in high risk for psychosis^46^. The right hippocampus seems vulnerable in high-risk young patients with only risk symptoms^46^. Thus, enlargement of the left hippocampus volume in adolescents with SPEs, who have not yet reached a diagnostic level, may reflect changes as compensation to prevent progression to a high-risk state. Further research will be needed to elucidate the detailed mechanism.

We revealed no significant differences in bilateral pallidal volumes between adolescents with SPEs and those without (Fig. 1, Supplementary Tables 6–10). Our previous results of bilateral pallidal enlargement in chronic schizophrenia were not replicated^7^. Previous MRI studies demonstrated enlarged pallidum volume in chronic/previously treated schizophrenia^7,27,47–51^, but normal pallidal volume in drug-naive schizophrenia^48,52^ and first-episode schizophrenia^50^. Consequently, our current results are mostly concordant with these previous studies. Left pallidal volumes are marginally significantly larger in adolescents with SPEs (Supplementary Tables 7–9). We suggest that subtle volumetric alterations in the left pallidum may already occur as a predisposing factor for developing psychosis.

Pallidal volume demonstrated leftward asymmetries in adolescents with SPEs and no significant (but marginally leftward) asymmetries in those without (Fig. 2, Supplementary Table 11). Our previous study revealed no asymmetry of pallidal volume in healthy controls but a significant leftward asymmetry in patients with schizophrenia (mean age: 35 years old)^7^. We suggest a slight leftward asymmetry for pallidum volume will disappear over time in healthy individuals, while a strong leftward asymmetry will remain or amplify in individuals who develop schizophrenia. Moreover, adolescents with SPEs showed significantly more leftward laterality of pallidal volume than those without (Fig. 3, Supplementary Tables 12–15). Our previous study revealed almost the same finding by comparing patients with schizophrenia and healthy subjects^7^. Thus, by overcoming the limitations of our previous results, we suggest that a leftward alteration of pallidal volume laterality should be considered possible premorbid neuropathology in schizophrenia. Previous studies reported left side-specific pallidal predisposing abnormalities in schizophrenia, such as an association between left pallidal volume and Disrupted in schizophrenia 1 (DISC1) single-nucleotide polymorphism (SNP) rs16854756^53^, and a higher-than-normal blood flow in the left globus pallidus in never-medicated patients with schizophrenia^54^. These findings support our suggestion that a leftward alteration of pallidal volume laterality should be a premorbid predisposition of schizophrenia.

In addition, we demonstrated marginally significantly larger volume in left pallidum and more leftward asymmetry for pallidal volume in boys with SPEs than those without, but no significant differences between girls with SPEs and those without. Moreover, by limiting the scope to Tanner stage 1, we revealed a larger volume in the left pallidum and more leftward asymmetry for pallidal volume in adolescents with SPEs than those without. The volumes of subcortical structures including the pallidum are likely to be affected by age^55^, sex^56^, and pubertal development^32^, which our data partially support (Supplementary Tables 2–4). Boys are less sexually developed than girls in early adolescence. This may be why the results obtained from the whole sample were replicated in only boys but not girls. Our current finding that limiting the scope to Tanner stage 1 strengthened statistical significance should support the theory of the effect of pubertal development on subcortical volumes^32^. Detailed mechanisms are expected to be uncovered in the future.

Impaired cortico-striato-thalamo-cortical loops are a prominent theory of the psychosis spectrum^57–59^. The globus pallidus is the main output structure from the striatum within that network, which involves motor, associative, and limbic functions^60,61^. The conventional theory suggests that the inhibitory output from the globus pallidus to the cortex is via the thalamus^62^. However, a direct projection from the globus pallidus externus to the frontal cortex was recently found in mice, which was only ipsilateral and sensitive to antipsychotics^63^. Thus, it may be implied that aberrant laterality exists in neural circuits and connectivity patterns of the globus pallidus in adolescents with psychotic experiences. Besides, interhemispheric functional connectivity within the globus pallidus is reduced in first-episode schizophrenia^64^, which may support that implication. However, detailed mechanisms of the association between pallidal volume laterality and psychotic experiences are yet to be seen. Further research is required to elucidate this phenomenon.

The current study has several limitations. First, the APSS data were acquired, on average, 16 months prior to MRI scanning. Thus, because of a relatively large time gap, the reliability of our results cannot be fully guaranteed. Age-related changes in SPEs or pallidal volume were not found within this study sample, and intra-individual longitudinal changes in these factors within such a narrow age range are therefore assumed to be small. Thus, the issue of time intervals will not be especially problematic in our current analyses. Second, this study, part of the first wave of the pn-TTC study, is a cross-sectional study, and, because of this, we cannot currently conclude causality between SPEs and pallidal volumetric alterations. We have already launched a longitudinal survey of the pn-TTC study to observe trajectory changes. Thus, this limitation will be overcome in the near future.

In conclusion, the current results of our “population neuroscience” study with a minimally biased, large-scale sample will provide new insights into the predisposing neuropathology of schizophrenia.

## Electronic supplementary material

Supplemental Material
