# Supplementary material for: Abnormal asymmetries in subcortical brain volume in early adolescents with subclinical psychotic experiences
Source: Transl Psychiatry. 2018 Nov 28;8:254. doi: 10.1038/s41398-018-0312-6 (PMC6261944; doi:10.1038/s41398-018-0312-6)
Supplement: Supplementary file 1 — Supplemental Material [file 41398_2018_312_MOESM1_ESM.docx]

**Supplementary Information**

**Abnormal asymmetries in subcortical brain volume in early adolescents with subclinical psychotic experiences**

Naohiro Okada^1, 2^, Noriaki Yahata^1, 3^, Daisuke Koshiyama^1^, Kentaro Morita^1^, Kingo Sawada^1^, Sho Kanata^1, 4^, Shinya Fujikawa^1^, Noriko Sugimoto^1^, Rie Toriyama^1^, Mio Masaoka^1^, Shinsuke Koike^1, 2, 5^, Tsuyoshi Araki^1^, Yukiko Kano^6^, Kaori Endo^7^, Syudo Yamasaki^7^, Shuntaro Ando^1, 7^, Atsushi Nishida^7^, Mariko Hiraiwa-Hasegawa^8^, Kiyoto Kasai^1, 2^

1. Department of Neuropsychiatry, Graduate School of Medicine, The University of Tokyo, Tokyo, Japan
2. International Research Center for Neurointelligence (WPI-IRCN), The University of Tokyo Institutes for Advanced Study (UTIAS), The University of Tokyo, Tokyo, Japan
3. Department of Molecular Imaging and Theranostics, National Institute of Radiological Sciences, National Institutes for Quantum and Radiological Science and Technology, Chiba, Japan
4. Department of Psychiatry, Teikyo University School of Medicine, Tokyo, Japan
5. UTokyo Institute for Diversity and Adaptation of Human Mind (UTIDAHM), The University of Tokyo, Tokyo, Japan
6. Department of Child Psychiatry, Graduate School of Medicine, The University of Tokyo, Tokyo, Japan
7. Department of Psychiatry and Behavioral Sciences, Tokyo Metropolitan Institute of Medical Science, Tokyo, Japan
8. Department of Evolutionary Studies of Biosystems, School of Advanced Sciences, The Graduate University for Advanced Studies (SOKENDAI), Kanagawa, Japan

**TABLE OF CONTENTS**

SUPPLEMENTARY METHODS …………………………………...…….6

Supplementary Method 1 …………………………………...…….6

Supplementary Method 2 …………………………………...…….8

Supplementary Method 3 …………………………………...…….11

Supplementary Method 4 …………………………………...…….13

SUPPLEMENTARY REFERENCES …………………………………...…….14

SUPPLEMENTARY TABLES …………………………………...…….17

Supplementary Table 1 …………………………………...…….17

Supplementary Table 2 …………………………………...…….18

Supplementary Table 3 …………………………………...…….19

Supplementary Table 4 …………………………………...…….20

Supplementary Table 5 …………………………………...…….21

Supplementary Table 6 …………………………………...…….22

Supplementary Table 7 …………………………………...…….23

Supplementary Table 8 …………………………………...…….24

Supplementary Table 9 …………………………………...…….25

Supplementary Table 10 …………………………………...…….26

Supplementary Table 11 …………………………………...…….27

Supplementary Table 12 …………………………………...…….28

Supplementary Table 13 …………………………………...…….29

Supplementary Table 14 …………………………………...…….30

Supplementary Table 15 …………………………………...…….31

Supplementary Table 16 …………………………………...…….32

Supplementary Table 17 …………………………………...…….33

Supplementary Table 18 …………………………………...…….34

Supplementary Table 19 …………………………………...…….35

Supplementary Table 20 …………………………………...…….36

Supplementary Table 21 …………………………………...…….37

Supplementary Table 22 …………………………………...…….38

Supplementary Table 23 …………………………………...…….39

Supplementary Table 24 …………………………………...…….40

Supplementary Table 25 …………………………………...…….41

SUPPLEMENTARY FIGURE …………………………………...…….42

Supplementary Figure 1 …………………………………...…….42

**SUPPLEMENTARY METHODS**

**Supplementary Method 1** Participants and ethics

This current study was conducted as part of the population-neuroscience study of the Tokyo TEEN Cohort (TTC, http://ttcp.umin.jp/) (pn-TTC), in which 301 early adolescents took part and biological markers including magnetic resonance imaging (MRI), deoxyribonucleic acid (DNA) methylation, stress hormones, and gonadal hormones were measured. The participants in the pn-TTC study were recruited from a larger participant group of the TTC project, a large-scale population-based cohort in the Tokyo metropolitan area, in which 3,171 early adolescents had taken part. Among the participants in the TTC project, those who demonstrated an interest in the pn-TTC study were regarded as candidate participants. The included subjects took part in the pn-TTC study approximately one year after their participation in the TTC study. Written informed consent was acquired from each participant and the participant’s primary parent before participation. All protocols were approved by the research ethics committees of the University of Tokyo Faculty of Medicine (approval no. 3150, 10057, and 10069), Tokyo Metropolitan Institute of Medical Science (approval no. 12–35), and the Graduate University for Advanced Studies (SOKENDAI) (approval no. 2012002). During their first visit, participants entered a mock scanner in order to acclimate to the MRI scanner environment and to practice lying still during scanning. During their second visit, participants underwent MRI scanning. The exclusion criteria for participation were as follows: (a) evident psychiatric or neurological disorder (e.g., down syndrome, epilepsy, autism spectrum disorder, or attention-deficit/hyperactivity disorder); (b) auditory or visual impairment (except myopia); (c) endocrinological disease, or disease that might have an effect on hypothalamic-pituitary-adrenal axis function (e.g., thyroid disease, diabetes mellitus, or renal dysfunction), adrenal dysfunction, or gonadotropic dysfunction; (d) recent or long-term use of medication that might influence the central nervous system (e.g., antihistamines and steroid hormones); (e) history of head trauma followed by loss of consciousness for five minutes or more; (f) metal implants (except titanium) in the body; and (g) failure to remain still during the MRI practice session in the first visit.

**Supplementary Method 2** Scanning and image preprocessing

**Image acquisition**

MRI scanning was performed on a Philips Achieva 3T system (Philips Medical Systems, Best, The Netherlands) using an eight-channel receive head coil. Each subject underwent an MRI examination, comprising fluid attenuated inversion recovery (FLAIR), a T1 three-dimensional (3D) magnetization-prepared rapid gradient echo sequence (3D-MPRAGE), MR angiography (MRA) sequences.

Sagittal T1-weighted images were acquired with the following parameters; repetition time (TR)  = 7.0 ms, echo time (TE) = 3.2 ms, flip angle = 9 degrees, matrix = 256 × 256, field of view (FOV) = 256 mm × 240 mm × 200 mm, voxel size = 1 mm × 1 mm × 1 mm, slice thickness = 1 mm, number of slices = 200. Total acquisition time was approximately 10 minutes and 42 seconds.

**Quality control of MRI images**

In the quality control step, original T1-weighted images were checked by visual inspection. We excluded images with insufficient brain coverage (FOV problem) and those with low signal-to-noise ratios or any artifacts (e.g., motion artifacts and magnetic susceptibility artifacts). Moreover, we excluded images with any abnormal organic findings (e.g., large arachnoid cysts and cavum septum pellucidum), which were found in T1-weighted, FLAIR, or MRA images.

**Image preprocessing**

T1-weighted imaging data that had passed the quality control step were processed with FreeSurfer software version 5.3 (http://surfer.nmr.mgh.harvard.edu)^1^ as described previously.^2,3^ FreeSurfer provides an automated processing pipeline. Through this procedure, we obtained images of subcortical segmentation and regional volumes for the bilateral lateral ventricles, thalamus, caudate, putamen, globus pallidus, hippocampus, amygdala, and accumbens, as well as intracranial volume (ICV). Briefly, this method comprises five steps: an affine registration with MNI305 space, an initial volumetric labeling, a B1 bias field correction, a high dimensional nonlinear volumetric alignment to the MNI305 atlas, and a volumetric labeling. FreeSurfer software was used for segmentation of adolescents’ brain images in some previous studies.^4,5^ Some images could not be processed for technical reasons during FreeSufer preprocessing. After the preprocessing, two independent researchers visually inspected each segmentation image in order to exclude images with poor parcellation.

**Supplementary Method 3** Psychological and physical evaluation

The 7-item Adolescent Psychotic-like Symptom Screener (APSS)^6^ is a self-administered questionnaire for screening subclinical psychotic experiences (SPEs) in adolescents. Three responses of “Yes, definitely,” “Maybe,” and “No, never” are possible, and scored as 1 point, 0.5 point, and 0 point, respectively. A total score is calculated by adding up the scores for all questionnaires, and its possible range is from 0 to 7. A total score of 2 or more is regarded as being SPE-positive, and the reliability of this cutoff point was validated by a previous study.^6^ Additionally, the APSS was used in some previous neuroimaging studies.^7,8^ Participants had already completed the APSS at the time of their participation in the TTC study. The mean interval duration between the psychological assessment by APSS and MRI scanning was 16 months (ranging from 7 to 39 months).

The Tanner stage is a scale to determine what stage of puberty an adolescent is currently in.^9-11^ Tanner stages range from 1 (prepubertal) to 5 (fully matured). In this study, primary parents, most of whom were mothers, rated their children’s Tanner stage according to pubic hair development, as well as to genital development (boys) or breast development (girls). We used the maximum of the two kinds of Tanner stage in our following analyses. Some children’s Tanner stages were determined prior to MRI scanning, while the others’ stages were determined on the same day as MRI scanning. The mean interval duration between the physical assessment by the Tanner stage and MRI scanning was 6 days (ranging from 0 to 141 days).

**Supplementary Method 4** Subject selection

Out of the 301 participants of the pn-TTC study, a total of 272 early adolescents (144 boys and 128 girls) with a mean age of 11.5 years underwent T1-weighted MRI scan. We identified inadequate images in the quality control of MRI images, and 63 subjects (36 boys and 27 girls) were excluded. A total of 209 adolescents (108 boys and 101 girls) remained. Through the FreeSurfer processing, one subject was excluded due to the incompletion of FreeSurfer processing, and no subject was excluded due to poor segmentation. A total of 208 adolescents (108 boys and 100 girls) remained. Five adolescents (2 boys and 3 girls) did not respond to the APSS questionnaire. Finally, 203 adolescents (106 boys and 97 girls) with a mean age of 11.6 years (range: 10.5 - 13.3 years) were included in the current study.**SUPPLEMENTARY REFERENCES**

1. Fischl B, Salat DH, Busa E, Albert M, Dieterich M, Haselgrove C *et al.* Whole brain segmentation: automated labeling of neuroanatomical structures in the human brain. *Neuron* 2002; **33:** 341-355.

2. van Erp TG, Hibar DP, Rasmussen JM, Glahn DC, Pearlson GD, Andreassen OA *et al.* Subcortical brain volume abnormalities in 2028 individuals with schizophrenia and 2540 healthy controls via the ENIGMA consortium. *Mol Psychiatry* 2016; **21:** 547-553.

3. Okada N, Fukunaga M, Yamashita F, Koshiyama D, Yamamori H, Ohi K *et al.* Abnormal asymmetries in subcortical brain volume in schizophrenia. *Mol Psychiatry* 2016; **21:** 1460-1466.

4. Juuhl-Langseth M, Rimol LM, Rasmussen IA, Jr., Thormodsen R, Holmen A, Emblem KE *et al.* Comprehensive segmentation of subcortical brain volumes in early onset schizophrenia reveals limited structural abnormalities. *Psychiatry Res* 2012; **203:** 14-23.

5. Dennison M, Whittle S, Yucel M, Vijayakumar N, Kline A, Simmons J *et al.* Mapping subcortical brain maturation during adolescence: evidence of hemisphere- and sex-specific longitudinal changes. *Dev Sci* 2013; **16:** 772-791.

6. Kelleher I, Harley M, Murtagh A, Cannon M. Are screening instruments valid for psychotic-like experiences? A validation study of screening questions for psychotic-like experiences using in-depth clinical interview. *Schizophr Bull* 2011; **37:** 362-369.

7. Jacobson S, Kelleher I, Harley M, Murtagh A, Clarke M, Blanchard M *et al.* Structural and functional brain correlates of subclinical psychotic symptoms in 11-13 year old schoolchildren. *Neuroimage* 2010; **49:** 1875-1885.

8. Jacobson McEwen SC, Connolly CG, Kelly AM, Kelleher I, O'Hanlon E, Clarke M *et al.* Resting-state connectivity deficits associated with impaired inhibitory control in non-treatment-seeking adolescents with psychotic symptoms. *Acta Psychiatr Scand* 2014; **129:** 134-142.

9. Tanner JM. Normal growth and techniques of growth assessment. *Clin Endocrinol Metab* 1986; **15:** 411-451.

10. Marshall WA, Tanner JM. Variations in pattern of pubertal changes in girls. *Arch Dis Child* 1969; **44:** 291-303.

11. Marshall WA, Tanner JM. Variations in the pattern of pubertal changes in boys. *Arch Dis Child* 1970; **45:** 13-23.

**SUPPLEMENTARY TABLES**

**Supplementary Table 1** Sex and age differences between the subclinical psychotic experience (SPE)-positive and SPE-negative groups.

|  | Overall | | SPE-negative | | SPE-positive | | *t/χ^2^* | *p* |
| --- | --- | --- | --- | --- | --- | --- | --- | --- |
|  | Mean | SD | Mean | SD | Mean | SD |  |  |
| N (boys/girls) ^a^ | 203 (106/97) | | 139 (72/67) | | 64 (34/30) | | 0.03 | 0.86 |
| age ^b^ | 11.6 | 0.7 | 11.5 | 0.7 | 11.6 | 0.8 | -0.65 | 0.52 |

1. A *χ^2^* test was conducted to assess sex differences.
2. A *t*-test was conducted to assess sex differences.

Abbreviations: SPE, subclinical psychotic experience.

**Supplementary Table 2** Sex differences in subcortical regional volumes.

|  | *t* value | *df* | *p* value |  | *t* value | *df* | *p* value |
| --- | --- | --- | --- | --- | --- | --- | --- |
| LLatVent | 1.14 | 201 | 0.26 | RLatVent | 0.49 | 201 | 0.62 |
| Lthal | 5.30 | 201 | 3.1 × 10^-7^ | Rthal | 6.72 | 201 | 1.8 × 10^-10^ |
| Lcaud | 1.21 | 201 | 0.23 | Rcaud | 0.35 | 197.41 | 0.73 |
| Lput | 5.03 | 199.00 | 1.1 × 10^-6^ | Rput | 6.79 | 199.84 | 1.2 × 10^-10^ |
| Lpal | 3.53 | 201 | 5.2 × 10^-4^ | Rpal | 4.33 | 201 | 2.4 × 10^-5^ |
| Lhippo | 5.12 | 201 | 7.2 × 10^-7^ | Rhippo | 5.69 | 201 | 4.5 × 10^-8^ |
| Lamyg | 5.77 | 201 | 3.0 × 10^-8^ | Ramyg | 7.41 | 201 | 3.3 × 10^-12^ |
| Laccumb | 2.21 | 201 | 2.8 × 10^-2^ | Raccumb | 3.94 | 201 | 1.1 × 10^-4^ |

For all subcortical regions, boys showed larger volumes than girls.

Abbreviations: df, degree of freedom; LLatVent, left lateral ventricle; RLatVent, right lateral ventricle; Lthal, left thalamus; Rthal, right thalamus; Lcaud, left caudate; Rcaud, right caudate; Lput, left putamen; Rput, right putamen; Lpal, left pallidum; Rpal, right pallidum; Lhippo, left hippocampus; Rhippo, right hippocampus; Lamyg, left amygdala; Ramyg, right amygdala; Laccumb, left accumbens; Raccumb, right accumbens.

**Supplementary Table 3** Age associations of subcortical regional volumes.

|  | Spearman’s *ρ* | *p* value |  | Spearman’s *ρ* | *p* value |
| --- | --- | --- | --- | --- | --- |
| LLatVent | 0.06 | 0.41 | RLatVent | 0.05 | 0.48 |
| Lthal | 0.10 | 0.16 | Rthal | 0.07 | 0.35 |
| Lcaud | 0.02 | 0.80 | Rcaud | -0.04 | 0.57 |
| Lput | -0.09 | 0.19 | Rput | -0.14 | 4.5 × 10^-2^ |
| Lpal | 0.01 | 0.92 | Rpal | 0.03 | 0.70 |
| Lhippo | 0.04 | 0.60 | Rhippo | 0.10 | 0.17 |
| Lamyg | -0.03 | 0.70 | Ramyg | -0.02 | 0.73 |
| Laccumb | -0.10 | 0.14 | Raccumb | -0.19 | 7.3 × 10^-3^ |

Abbreviations: LLatVent, left lateral ventricle; RLatVent, right lateral ventricle; Lthal, left thalamus; Rthal, right thalamus; Lcaud, left caudate; Rcaud, right caudate; Lput, left putamen; Rput, right putamen; Lpal, left pallidum; Rpal, right pallidum; Lhippo, left hippocampus; Rhippo, right hippocampus; Lamyg, left amygdala; Ramyg, right amygdala; Laccumb, left accumbens; Raccumb, right accumbens.

**Supplementary Table 4** Correlations between Tanner stages and subcortical regional volumes.

|  | Spearman’s *ρ* | *p* value |  | Spearman’s *ρ* | *p* value |
| --- | --- | --- | --- | --- | --- |
| LLatVent | 0.09 | 0.21 | RLatVent | 0.09 | 0.23 |
| Lthal | 0.04 | 0.55 | Rthal | 0.02 | 0.78 |
| Lcaud | 0.05 | 0.46 | Rcaud | 0.02 | 0.79 |
| Lput | -0.14 | 4.3 × 10^-2^ | Rput | -0.24 | 7.4 × 10^-4^ |
| Lpal | 0.02 | 0.75 | Rpal | -0.02 | 0.80 |
| Lhippo | 0.06 | 0.38 | Rhippo | 0.05 | 0.47 |
| Lamyg | 0.01 | 0.87 | Ramyg | 0.02 | 0.77 |
| Laccumb | -0.23 | 1.3 × 10^-3^ | Raccumb | -0.07 | 0.31 |

Data on Tanner stages are missing for three adolescents.

Abbreviations: LLatVent, left lateral ventricle; RLatVent, right lateral ventricle; Lthal, left thalamus; Rthal, right thalamus; Lcaud, left caudate; Rcaud, right caudate; Lput, left putamen; Rput, right putamen; Lpal, left pallidum; Rpal, right pallidum; Lhippo, left hippocampus; Rhippo, right hippocampus; Lamyg, left amygdala; Ramyg, right amygdala; Laccumb, left accumbens; Raccumb, right accumbens.

**Supplementary Table 5** Means and standard deviations of subcortical volumes (mm^3^) in the subclinical psychotic experience (SPE)-negative and SPE-positive groups.

|  | SPE-negative | | SPE-positive | |  | SPE-negative | | SPE-positive | |
| --- | --- | --- | --- | --- | --- | --- | --- | --- | --- |
|  | Mean (mm^3^) | SD (mm^3^) | Mean (mm^3^) | SD (mm^3^) |  | Mean (mm^3^) | SD (mm^3^) | Mean (mm^3^) | SD (mm^3^) |
| LLatVent | 5120.4 | 2951.7 | 5691.7 | 3091.3 | RLatVent | 4400.7 | 2197.8 | 5220.3 | 2843.6 |
| Lthal | 8064.0 | 710.2 | 8103.9 | 656.4 | Rthal | 7168.9 | 607.2 | 7249.8 | 585.0 |
| Lcaud | 3876.6 | 525.3 | 4026.0 | 547.0 | Rcaud | 3870.7 | 538.2 | 4059.6 | 553.5 |
| Lput | 6360.6 | 736.3 | 6358.2 | 678.5 | Rput | 6341.8 | 672.1 | 6290.4 | 570.8 |
| Lpal | 1716.8 | 225.7 | 1775.8 | 208.1 | Rpal | 1682.8 | 173.0 | 1683.2 | 180.4 |
| Lhippo | 4389.0 | 350.6 | 4502.9 | 417.6 | Rhippo | 4557.2 | 396.2 | 4623.2 | 388.4 |
| Lamyg | 1789.8 | 185.7 | 1766.9 | 193.2 | Ramyg | 2066.9 | 254.6 | 2064.3 | 262.3 |
| Laccumb | 739.7 | 142.6 | 768.5 | 138.9 | Raccumb | 724.3 | 107.6 | 736.5 | 102.2 |

Abbreviations: SPE, subclinical psychotic experience; SD, standard deviation; LLatVent, left lateral ventricle; RLatVent, right lateral ventricle; Lthal, left thalamus; Rthal, right thalamus; Lcaud, left caudate; Rcaud, right caudate; Lput, left putamen; Rput, right putamen; Lpal, left pallidum; Rpal, right pallidum; Lhippo, left hippocampus; Rhippo, right hippocampus; Lamyg, left amygdala; Ramyg, right amygdala; Laccumb, left accumbens; Raccumb, right accumbens.

**Supplementary Table 6** Regression beta coefficients of a group dummy variable [subclinical psychotic experience (SPE)-negative = 0, SPE-positive = 1] to subcortical regional volumes controlling for age, sex and intracranial volume (ICV) (Model 1). The unit of beta and standard error (SE) is mm^3^.

|  | Beta (mm^3^) | SE (mm^3^) | Cohen's *d* | SE of Cohen's *d* | *t* value | *p* value |
| --- | --- | --- | --- | --- | --- | --- |
| Lhippo | 80.35 | 48.09 | 0.21 | 0.13 | 1.67 | 0.096 |
| Rhippo | 24.80 | 48.10 | 0.06 | 0.12 | 0.52 | 0.607 |
| Lamyg | -37.32 | 23.97 | -0.20 | 0.13 | -1.56 | 0.121 |
| Ramyg | -23.27 | 31.56 | -0.09 | 0.12 | -0.74 | 0.462 |
| Lthal | -41.19 | 81.34 | -0.06 | 0.12 | -0.51 | 0.613 |
| Rthal | 10.94 | 67.83 | 0.02 | 0.11 | 0.16 | 0.872 |
| Laccumb | 20.57 | 19.50 | 0.15 | 0.14 | 1.05 | 0.293 |
| Raccumb | 6.98 | 14.48 | 0.07 | 0.14 | 0.48 | 0.630 |
| Lcaud | 94.46 | 68.71 | 0.18 | 0.13 | 1.37 | 0.171 |
| Rcaud | 141.02 | 72.56 | 0.26 | 0.13 | 1.94 | 0.053 |
| Lput | -48.72 | 96.53 | -0.08 | 0.15 | -0.50 | 0.614 |
| Rput | -95.46 | 79.32 | -0.15 | 0.12 | -1.20 | 0.230 |
| Lpal | 45.70 | 31.16 | 0.21 | 0.14 | 1.47 | 0.144 |
| Rpal | -12.93 | 23.87 | -0.07 | 0.14 | -0.54 | 0.589 |
| LLatVent | 337.25 | 420.05 | 0.11 | 0.14 | 0.80 | 0.423 |
| RLatVent | 663.28 | 345.32 | 0.27 | 0.14 | 1.92 | 0.056 |

Abbreviations: SE, standard error; Lhippo, left hippocampus; Rhippo, right hippocampus; Lamyg, left amygdala; Ramyg, right amygdala; Lthal, left thalamus; Rthal, right thalamus; Laccumb, left accumbens; Raccumb, right accumbens; Lcaud, left caudate; Rcaud, right caudate; Lput, left putamen; Rput, right putamen; Lpal, left pallidum; Rpal, right pallidum; LLatVent, left lateral ventricle; RLatVent, right lateral ventricle.

**Supplementary Table 7** Regression beta coefficients of a group dummy variable [subclinical psychotic experience (SPE)-negative = 0, SPE-positive = 1] to subcortical regional volumes without covariates (Model 2). The unit of beta and standard error (SE) is mm^3^.

|  | Beta (mm^3^) | SE (mm^3^) | Cohen's *d* | SE of Cohen's *d* | *t* value | *p* value |
| --- | --- | --- | --- | --- | --- | --- |
| Lhippo | 113.98 | 56.32 | 0.30 | 0.15 | 2.02 | 0.044 |
| Rhippo | 66.01 | 59.48 | 0.17 | 0.15 | 1.11 | 0.268 |
| Lamyg | -22.93 | 28.41 | -0.12 | 0.15 | -0.81 | 0.421 |
| Ramyg | -2.60 | 38.83 | -0.01 | 0.15 | -0.07 | 0.947 |
| Lthal | 39.91 | 104.81 | 0.06 | 0.15 | 0.38 | 0.704 |
| Rthal | 80.92 | 90.68 | 0.13 | 0.15 | 0.89 | 0.373 |
| Laccumb | 28.79 | 21.37 | 0.20 | 0.15 | 1.35 | 0.179 |
| Raccumb | 12.16 | 16.00 | 0.11 | 0.15 | 0.76 | 0.448 |
| Lcaud | 149.39 | 80.39 | 0.28 | 0.15 | 1.86 | 0.065 |
| Rcaud | 188.89 | 82.03 | 0.34 | 0.15 | 2.30 | 0.022 |
| Lput | -2.37 | 108.56 | 0.00 | 0.17 | -0.02 | 0.983 |
| Rput | -51.43 | 96.99 | -0.08 | 0.15 | -0.53 | 0.596 |
| Lpal | 59.00 | 33.29 | 0.27 | 0.15 | 1.77 | 0.078 |
| Rpal | 0.41 | 26.49 | 0.00 | 0.15 | 0.02 | 0.988 |
| LLatVent | 571.34 | 452.60 | 0.19 | 0.15 | 1.26 | 0.208 |
| RLatVent | 819.65 | 365.39 | 0.34 | 0.15 | 2.24 | 0.026 |

Abbreviations: SE, standard error; Lhippo, left hippocampus; Rhippo, right hippocampus; Lamyg, left amygdala; Ramyg, right amygdala; Lthal, left thalamus; Rthal, right thalamus; Laccumb, left accumbens; Raccumb, right accumbens; Lcaud, left caudate; Rcaud, right caudate; Lput, left putamen; Rput, right putamen; Lpal, left pallidum; Rpal, right pallidum; LLatVent, left lateral ventricle; RLatVent, right lateral ventricle.

**Supplementary Table 8** Regression beta coefficients of a group dummy variable [subclinical psychotic experience (SPE)-negative = 0, SPE-positive = 1] to subcortical regional volumes controlling for the effect of age (Model 3). The unit of beta and standard error (SE) is mm^3^.

|  | Beta (mm^3^) | SE (mm^3^) | Cohen's *d* | SE of Cohen's *d* | *t* value | *p* value |
| --- | --- | --- | --- | --- | --- | --- |
| Lhippo | 110.99 | 56.33 | 0.30 | 0.15 | 1.97 | 0.050 |
| Rhippo | 60.95 | 59.17 | 0.15 | 0.15 | 1.03 | 0.304 |
| Lamyg | -22.15 | 28.49 | -0.12 | 0.15 | -0.78 | 0.438 |
| Ramyg | -3.20 | 38.95 | -0.01 | 0.15 | -0.08 | 0.935 |
| Lthal | 30.63 | 104.19 | 0.04 | 0.15 | 0.29 | 0.769 |
| Rthal | 71.36 | 89.78 | 0.12 | 0.15 | 0.79 | 0.428 |
| Laccumb | 30.75 | 21.22 | 0.22 | 0.15 | 1.45 | 0.149 |
| Raccumb | 13.74 | 15.87 | 0.13 | 0.15 | 0.87 | 0.388 |
| Lcaud | 146.90 | 80.58 | 0.27 | 0.15 | 1.82 | 0.070 |
| Rcaud | 189.72 | 82.31 | 0.35 | 0.15 | 2.30 | 0.022 |
| Lput | 0.72 | 108.84 | 0.00 | 0.17 | 0.01 | 0.995 |
| Rput | -44.96 | 96.81 | -0.07 | 0.15 | -0.46 | 0.643 |
| Lpal | 58.68 | 33.40 | 0.26 | 0.15 | 1.76 | 0.080 |
| Rpal | -0.77 | 26.52 | 0.00 | 0.15 | -0.03 | 0.977 |
| LLatVent | 555.61 | 453.55 | 0.19 | 0.15 | 1.23 | 0.222 |
| RLatVent | 823.33 | 366.63 | 0.34 | 0.15 | 2.25 | 0.026 |

Abbreviations: SE, standard error; Lhippo, left hippocampus; Rhippo, right hippocampus; Lamyg, left amygdala; Ramyg, right amygdala; Lthal, left thalamus; Rthal, right thalamus; Laccumb, left accumbens; Raccumb, right accumbens; Lcaud, left caudate; Rcaud, right caudate; Lput, left putamen; Rput, right putamen; Lpal, left pallidum; Rpal, right pallidum; LLatVent, left lateral ventricle; RLatVent, right lateral ventricle.

**Supplementary Table 9** Regression beta coefficients of a group dummy variable [subclinical psychotic experiences (SPE)-negative = 0, SPE-positive = 1] to subcortical regional volumes controlling for the effect of age and sex (Model 4). The unit of beta and standard error (SE) is mm^3^.

|  | Beta (mm^3^) | SE (mm^3^) | Cohen's *d* | SE of Cohen's *d* | *t* value | *p* value |
| --- | --- | --- | --- | --- | --- | --- |
| Lhippo | 108.48 | 53.16 | 0.29 | 0.14 | 2.04 | 0.043 |
| Rhippo | 58.07 | 55.17 | 0.15 | 0.14 | 1.05 | 0.294 |
| Lamyg | -23.59 | 26.39 | -0.13 | 0.14 | -0.89 | 0.372 |
| Ramyg | -5.58 | 34.61 | -0.02 | 0.13 | -0.16 | 0.872 |
| Lthal | 25.89 | 98.05 | 0.04 | 0.14 | 0.26 | 0.792 |
| Rthal | 66.33 | 81.51 | 0.11 | 0.14 | 0.81 | 0.417 |
| Laccumb | 30.29 | 20.99 | 0.21 | 0.15 | 1.44 | 0.150 |
| Raccumb | 13.15 | 15.27 | 0.12 | 0.14 | 0.86 | 0.390 |
| Lcaud | 146.03 | 80.52 | 0.27 | 0.15 | 1.81 | 0.071 |
| Rcaud | 189.46 | 82.50 | 0.35 | 0.15 | 2.30 | 0.023 |
| Lput | -4.11 | 102.79 | -0.01 | 0.16 | -0.04 | 0.968 |
| Rput | -50.59 | 87.13 | -0.08 | 0.14 | -0.58 | 0.562 |
| Lpal | 57.62 | 32.50 | 0.26 | 0.15 | 1.77 | 0.078 |
| Rpal | -1.78 | 25.46 | -0.01 | 0.15 | -0.07 | 0.944 |
| LLatVent | 551.06 | 453.39 | 0.18 | 0.15 | 1.22 | 0.226 |
| RLatVent | 821.66 | 367.35 | 0.34 | 0.15 | 2.24 | 0.026 |

Abbreviations: SE, standard error; Lhippo, left hippocampus; Rhippo, right hippocampus; Lamyg, left amygdala; Ramyg, right amygdala; Lthal, left thalamus; Rthal, right thalamus; Laccumb, left accumbens; Raccumb, right accumbens; Lcaud, left caudate; Rcaud, right caudate; Lput, left putamen; Rput, right putamen; Lpal, left pallidum; Rpal, right pallidum; LLatVent, left lateral ventricle; RLatVent, right lateral ventricle.

**Supplementary Table 10** Regression beta coefficients of a group dummy variable [subclinical psychotic experience (SPE)-negative = 0, SPE-positive = 1] to subcortical regional volumes controlling for the effect of age, sex, intracranial volume (ICV), and Tanner stage (Model 5). The unit of beta and standard error (SE) is mm^3^.

|  | Beta (mm^3^) | SE (mm^3^) | Cohen's *d* | SE of Cohen's *d* | *t* value | *p* value |
| --- | --- | --- | --- | --- | --- | --- |
| Lhippo | 75.00 | 48.85 | 0.20 | 0.13 | 1.54 | 0.126 |
| Rhippo | 12.83 | 48.55 | 0.03 | 0.12 | 0.26 | 0.792 |
| Lamyg | -41.94 | 24.15 | -0.22 | 0.13 | -1.74 | 0.084 |
| Ramyg | -29.56 | 31.89 | -0.12 | 0.12 | -0.93 | 0.355 |
| Lthal | -49.20 | 82.75 | -0.07 | 0.12 | -0.59 | 0.553 |
| Rthal | 9.80 | 68.92 | 0.02 | 0.11 | 0.14 | 0.887 |
| Laccumb | 28.83 | 19.34 | 0.20 | 0.14 | 1.49 | 0.138 |
| Raccumb | 11.07 | 14.49 | 0.10 | 0.14 | 0.76 | 0.446 |
| Lcaud | 88.53 | 69.72 | 0.17 | 0.13 | 1.27 | 0.206 |
| Rcaud | 136.14 | 73.66 | 0.25 | 0.13 | 1.85 | 0.066 |
| Lput | -42.16 | 97.21 | -0.07 | 0.15 | -0.43 | 0.665 |
| Rput | -83.55 | 78.34 | -0.13 | 0.12 | -1.07 | 0.287 |
| Lpal | 49.47 | 31.39 | 0.22 | 0.14 | 1.58 | 0.117 |
| Rpal | -12.68 | 24.11 | -0.07 | 0.14 | -0.53 | 0.599 |
| LLatVent | 265.73 | 425.56 | 0.09 | 0.14 | 0.62 | 0.533 |
| RLatVent | 613.57 | 349.67 | 0.25 | 0.14 | 1.75 | 0.081 |

Data on Tanner stages are missing for three adolescents.

Abbreviations: SE, standard error; Lhippo, left hippocampus; Rhippo, right hippocampus; Lamyg, left amygdala; Ramyg, right amygdala; Lthal, left thalamus; Rthal, right thalamus; Laccumb, left accumbens; Raccumb, right accumbens; Lcaud, left caudate; Rcaud, right caudate; Lput, left putamen; Rput, right putamen; Lpal, left pallidum; Rpal, right pallidum; LLatVent, left lateral ventricle; RLatVent, right lateral ventricle.

**Supplementary Table 11** Means, standard deviations and one-sample *t*-test results for laterality indices (LIs) of subcortical volumes in the subclinical psychotic experience (SPE)-negative and SPE-positive groups.

|  | SPE-negative | | | | | SPE-positive | | | | |
| --- | --- | --- | --- | --- | --- | --- | --- | --- | --- | --- |
|  | Mean | SD | Cohen's *d* | *t* value | *p* value | Mean | SD | Cohen's *d* | *t* value | *p* value |
| LatVent | 0.064 | 0.144 | 0.45 | 5.32 | 7.6 × 10^-7^ | 0.044 | 0.114 | 0.38 | 3.04 | 3.4 × 10^-3^ |
| thal | 0.058 | 0.028 | 2.05 | 24.37 | 6.4 × 10^-52^ | 0.056 | 0.031 | 1.77 | 14.12 | 3.5 × 10^-21^ |
| caud | 0.001 | 0.042 | 0.02 | 0.19 | 0.76 | -0.004 | 0.04 | -0.11 | -0.84 | 0.40 |
| put | 0.001 | 0.029 | 0.03 | 0.41 | 0.70 | 0.005 | 0.034 | 0.14 | 1.09 | 0.28 |
| pal | 0.009 | 0.056 | 0.16 | 1.92 | 0.08 | 0.026 | 0.062 | 0.42 | 3.37 | 1.3 × 10^-3^ |
| hippo | -0.019 | 0.027 | -0.68 | -8.07 | 8.2 × 10^-13^ | -0.014 | 0.035 | -0.39 | -3.1 | 2.9 × 10^-3^ |
| amyg | -0.071 | 0.044 | -1.61 | -19.16 | 2.2 × 10^-40^ | -0.076 | 0.043 | -1.79 | -14.32 | 1.8 × 10^-21^ |
| accumb | 0.006 | 0.073 | 0.08 | 0.98 | 0.28 | 0.018 | 0.069 | 0.26 | 2.05 | 4.5 × 10^-2^ |

Abbreviations: SPE, subclinical psychotic experience; SD, standard deviation; LatVent, lateral ventricle; thal, thalamus; caud, caudate; put, putamen; pal, pallidum; hippo, hippocampus; amyg, amygdala; accumb, accumbens.

**Supplementary Table 12** Regression beta coefficients of a group dummy variable [subclinical psychotic experience (SPE)-negative = 0, SPE-positive = 1] to laterality indices (LIs) of subcortical regional volumes controlling for the effect of age and sex (Model 1).

|  | Beta | SE | Cohen's *d* | SE of Cohen's *d* | *t* value | *p* value |
| --- | --- | --- | --- | --- | --- | --- |
| thal | 0.00 | 0.00 | -0.10 | 0.15 | -0.65 | 0.516 |
| LatVent | -0.02 | 0.02 | -0.16 | 0.15 | -1.06 | 0.291 |
| caud | -0.01 | 0.01 | -0.14 | 0.15 | -0.93 | 0.352 |
| put | 0.00 | 0.00 | 0.12 | 0.15 | 0.76 | 0.446 |
| pal | 0.02 | 0.01 | 0.31 | 0.15 | 2.05 | 0.041 |
| accumb | 0.01 | 0.01 | 0.16 | 0.15 | 1.04 | 0.300 |
| amyg | -0.01 | 0.01 | -0.12 | 0.15 | -0.84 | 0.403 |
| hippo | 0.01 | 0.00 | 0.17 | 0.15 | 1.13 | 0.260 |

Abbreviations: SE, standard error; thal, thalamus; LatVent, lateral ventricle; caud, caudate; put, putamen; pal, pallidum; accumb, accumbens; amyg, amygdala; hippo, hippocampus.

**Supplementary Table 13** Regression beta coefficients of a group dummy variable [subclinical psychotic experience (SPE)-negative = 0, SPE-positive = 1] to laterality indices (LIs) of subcortical regional volumes without covariates (Model 2).

|  | Beta | SE | Cohen's *d* | SE of Cohen's *d* | *t* value | *p* value |
| --- | --- | --- | --- | --- | --- | --- |
| thal | 0.00 | 0.00 | -0.10 | 0.15 | -0.68 | 0.498 |
| LatVent | -0.02 | 0.02 | -0.15 | 0.15 | -0.98 | 0.328 |
| caud | -0.01 | 0.01 | -0.13 | 0.15 | -0.85 | 0.395 |
| put | 0.00 | 0.00 | 0.12 | 0.15 | 0.80 | 0.424 |
| pal | 0.02 | 0.01 | 0.31 | 0.15 | 2.03 | 0.043 |
| accumb | 0.01 | 0.01 | 0.15 | 0.15 | 1.01 | 0.315 |
| amyg | -0.01 | 0.01 | -0.13 | 0.15 | -0.89 | 0.377 |
| hippo | 0.00 | 0.00 | 0.16 | 0.15 | 1.08 | 0.282 |

Abbreviations: SE, standard error; thal, thalamus; LatVent, lateral ventricle; caud, caudate; put, putamen; pal, pallidum; accumb, accumbens; amyg, amygdala; hippo, hippocampus.

**Supplementary Table 14** Regression beta coefficients of a group dummy variable [subclinical psychotic experience (SPE)-negative = 0, SPE-positive = 1] to laterality indices (LIs) of subcortical regional volumes controlling for the effect of age (Model 3).

|  | Beta | SE | Cohen's *d* | SE of Cohen's *d* | *t* value | *p* value |
| --- | --- | --- | --- | --- | --- | --- |
| thal | 0.00 | 0.00 | -0.10 | 0.15 | -0.66 | 0.509 |
| LatVent | -0.02 | 0.02 | -0.16 | 0.15 | -1.04 | 0.302 |
| caud | -0.01 | 0.01 | -0.14 | 0.15 | -0.91 | 0.361 |
| put | 0.00 | 0.00 | 0.11 | 0.15 | 0.75 | 0.457 |
| pal | 0.02 | 0.01 | 0.31 | 0.15 | 2.06 | 0.041 |
| accumb | 0.01 | 0.01 | 0.16 | 0.15 | 1.03 | 0.303 |
| amyg | -0.01 | 0.01 | -0.13 | 0.15 | -0.85 | 0.396 |
| hippo | 0.01 | 0.00 | 0.17 | 0.15 | 1.12 | 0.262 |

Abbreviations: SE, standard error; thal, thalamus; LatVent, lateral ventricle; caud, caudate; put, putamen; pal, pallidum; accumb, accumbens; amyg, amygdala; hippo, hippocampus.

**Supplementary Table 15** Regression beta coefficients of a group dummy variable [subclinical psychotic experience (SPE)-negative = 0, SPE-positive = 1] to laterality indices (LIs) of subcortical regional volumes controlling for the effect of age, sex, and Tanner stage (Model 4).

|  | Beta | SE | Cohen's *d* | SE of Cohen's *d* | *t* value | *p* value |
| --- | --- | --- | --- | --- | --- | --- |
| thal | 0.00 | 0.00 | -0.11 | 0.15 | -0.74 | 0.461 |
| LatVent | -0.02 | 0.02 | -0.17 | 0.15 | -1.11 | 0.267 |
| caud | -0.01 | 0.01 | -0.14 | 0.15 | -0.93 | 0.355 |
| put | 0.00 | 0.00 | 0.10 | 0.15 | 0.65 | 0.514 |
| pal | 0.02 | 0.01 | 0.33 | 0.15 | 2.13 | 0.035 |
| accumb | 0.01 | 0.01 | 0.20 | 0.15 | 1.33 | 0.185 |
| amyg | -0.01 | 0.01 | -0.12 | 0.15 | -0.78 | 0.439 |
| hippo | 0.01 | 0.00 | 0.19 | 0.15 | 1.25 | 0.214 |

Data on Tanner stages are missing for three adolescents.

Abbreviations: SE, standard error; thal, thalamus; LatVent, lateral ventricle; caud, caudate; put, putamen; pal, pallidum; accumb, accumbens; amyg, amygdala; hippo, hippocampus.

**Supplementary Table 16** Regression beta coefficients of a group dummy variable [subclinical psychotic experience (SPE)-negative = 0, SPE-positive = 1] to pallidal volume controlling for the effect of age and intracranial volume (ICV) (Model 1); no covariates (Model 2); age (Model 3); and age, ICV, and Tanner stage (Model 4) in (a) boys and (b) girls.

(a)

|  | Beta (mm^3^) | SE (mm^3^) | Cohen's *d* | SE of Cohen's *d* | *t* value | *p* value |  |
| --- | --- | --- | --- | --- | --- | --- | --- |
| Lpal | 87.03 | 47.74 | 0.37 | 0.20 | 1.82 | 0.071 | Model 1 |
|  | 78.16 | 49.03 | 0.33 | 0.21 | 1.59 | 0.114 | Model 2 |
|  | 75.96 | 49.76 | 0.32 | 0.21 | 1.53 | 0.130 | Model 3 |
|  | 86.28 | 48.12 | 0.36 | 0.20 | 1.79 | 0.076 | Model 4 |
| Rpal | 9.30 | 35.21 | 0.05 | 0.20 | 0.26 | 0.792 | Model 1 |
|  | 7.98 | 36.63 | 0.05 | 0.21 | 0.22 | 0.828 | Model 2 |
|  | 0.77 | 36.86 | 0.00 | 0.21 | 0.02 | 0.983 | Model 3 |
|  | 13.99 | 35.51 | 0.08 | 0.20 | 0.39 | 0.694 | Model 4 |

(b)

|  | Beta (mm^3^) | SE (mm^3^) | Cohen's *d* | SE of Cohen's *d* | *t* value | *p* value |  |
| --- | --- | --- | --- | --- | --- | --- | --- |
| Lpal | 3.97 | 40.72 | 0.02 | 0.22 | 0.10 | 0.923 | Model 1 |
|  | 34.67 | 41.53 | 0.18 | 0.22 | 0.83 | 0.406 | Model 2 |
|  | 32.68 | 41.79 | 0.17 | 0.22 | 0.78 | 0.436 | Model 3 |
|  | 13.69 | 40.62 | 0.07 | 0.22 | 0.34 | 0.737 | Model 4 |
| Rpal | -43.31 | 33.21 | -0.27 | 0.21 | -1.30 | 0.195 | Model 1 |
|  | -10.88 | 35.12 | -0.07 | 0.22 | -0.31 | 0.757 | Model 2 |
|  | -12.56 | 35.35 | -0.08 | 0.22 | -0.36 | 0.723 | Model 3 |
|  | -48.51 | 33.66 | -0.30 | 0.21 | -1.44 | 0.153 | Model 4 |

Data on Tanner stages are missing for three adolescents.

Abbreviations: SE, standard error; Lpal, left pallidum; Rpal, right pallidum.

**Supplementary Table 17** Regression beta coefficients of a group dummy variable [subclinical psychotic experience (SPE)-negative = 0, SPE-positive = 1] to laterality indices (LIs) of pallidal volume controlling for the effect of age (Model 1); no covariates (Model 2); and age and Tanner stage (Model 3) in (a) boys and (b) girls.

(a)

| Beta | SE | Cohen's *d* | SE of Cohen's *d* | *t* value | *p* value |  |
| --- | --- | --- | --- | --- | --- | --- |
| 0.02 | 0.01 | 0.38 | 0.21 | 1.80 | 0.075 | Model 1 |
| 0.02 | 0.01 | 0.36 | 0.21 | 1.73 | 0.087 | Model 2 |
| 0.02 | 0.01 | 0.35 | 0.21 | 1.64 | 0.104 | Model 3 |

(b)

| Beta | SE | Cohen's *d* | SE of Cohen's *d* | *t* value | *p* value |  |
| --- | --- | --- | --- | --- | --- | --- |
| 0.01 | 0.01 | 0.24 | 0.22 | 1.07 | 0.286 | Model 1 |
| 0.01 | 0.01 | 0.24 | 0.22 | 1.10 | 0.275 | Model 2 |
| 0.02 | 0.01 | 0.32 | 0.22 | 1.44 | 0.155 | Model 3 |

Data on Tanner stages are missing for three adolescents.

Abbreviation: SE, standard error.

**Supplementary Table 18** Regression beta coefficients of a group dummy variable [subclinical psychotic experience (SPE)-negative = 0, SPE-positive = 1] to left and right pallidal volume controlling for the effect of age, sex, and intracranial volume (ICV) (Model 1); no covariates (Model 2); age (Model 3); and age and sex (Model 4) in adolescents in Tanner stage 1.

|  | Beta (mm^3^) | SE (mm^3^) | Cohen's *d* | SE of Cohen's *d* | *t* value | *p* value |  |
| --- | --- | --- | --- | --- | --- | --- | --- |
| Lpal | 174.16 | 65.41 | 0.80 | 0.30 | 2.66 | 0.011 | Model 1 |
|  | 152.11 | 70.09 | 0.70 | 0.32 | 2.17 | 0.035 | Model 2 |
|  | 162.48 | 68.86 | 0.75 | 0.32 | 2.36 | 0.023 | Model 3 |
|  | 170.68 | 68.32 | 0.79 | 0.31 | 2.50 | 0.016 | Model 4 |
| Rpal | -66.51 | 49.28 | -0.41 | 0.31 | -1.35 | 0.185 | Model 1 |
|  | -81.39 | 53.31 | -0.51 | 0.33 | -1.53 | 0.134 | Model 2 |
|  | -80.21 | 54.10 | -0.50 | 0.34 | -1.48 | 0.145 | Model 3 |
|  | -68.87 | 50.94 | -0.43 | 0.32 | -1.35 | 0.184 | Model 4 |

Abbreviations: SE, standard error; Lpal, left pallidum; Rpal, right pallidum.

**Supplementary Table 19** Regression beta coefficients of a group dummy variable [subclinical psychotic experience (SPE)-negative = 0, SPE-positive = 1] to laterality indices (LIs) of pallidal volume controlling for the effect of age and sex (Model 1); no covariates (Model 2); and age (Model 3) in adolescents in Tanner stage 1.

| Beta | SE | Cohen's *d* | SE of Cohen's *d* | *t* value | *p* value |  |
| --- | --- | --- | --- | --- | --- | --- |
| 0.07 | 0.02 | 1.06 | 0.30 | 3.53 | 1.0 × 10^-3^ | Model 1 |
| 0.07 | 0.02 | 1.04 | 0.30 | 3.43 | 1.3 × 10^-3^ | Model 2 |
| 0.07 | 0.02 | 1.08 | 0.30 | 3.63 | 7.5 × 10^-4^ | Model 3 |

Abbreviation: SE, standard error.

**Supplementary Table 20** Regression beta coefficients of a group dummy variable [subclinical psychotic experience (SPE)-negative = 0, SPE-positive = 1] to left and right pallidal volume controlling for the effect of age and intracranial volume (ICV) (Model 1); no covariates (Model 2); and age (Model 3) in (a) boys and (b) girls in Tanner stage 1.

(a)

|  | Beta (mm^3^) | SE (mm^3^) | Cohen's *d* | SE of Cohen's *d* | *t* value | *p* value |  |
| --- | --- | --- | --- | --- | --- | --- | --- |
| Lpal | 289.62 | 139.02 | 1.03 | 0.49 | 2.08 | 0.052 | Model 1 |
|  | 205.28 | 139.03 | 0.73 | 0.50 | 1.48 | 0.155 | Model 2 |
|  | 271.23 | 145.71 | 0.97 | 0.52 | 1.86 | 0.078 | Model 3 |
| Rpal | 30.11 | 77.85 | 0.20 | 0.53 | 0.39 | 0.703 | Model 1 |
|  | -19.37 | 76.99 | -0.13 | 0.52 | -0.25 | 0.804 | Model 2 |
|  | 21.56 | 79.76 | 0.15 | 0.54 | 0.27 | 0.790 | Model 3 |

(b)

|  | Beta (mm^3^) | SE (mm^3^) | Cohen's *d* | SE of Cohen's *d* | *t* value | *p* value |  |
| --- | --- | --- | --- | --- | --- | --- | --- |
| Lpal | 90.10 | 49.55 | 0.66 | 0.36 | 1.82 | 0.084 | Model 1 |
|  | 121.08 | 56.78 | 0.89 | 0.42 | 2.13 | 0.044 | Model 2 |
|  | 96.78 | 53.08 | 0.71 | 0.39 | 1.82 | 0.083 | Model 3 |
| Rpal | -113.17 | 68.21 | -0.73 | 0.44 | -1.66 | 0.113 | Model 1 |
|  | -112.79 | 66.57 | -0.73 | 0.43 | -1.69 | 0.104 | Model 2 |
|  | -107.23 | 69.28 | -0.70 | 0.45 | -1.55 | 0.137 | Model 3 |

Abbreviations: SE, standard error; Lpal, left pallidum; Rpal, right pallidum.

**Supplementary Table 21** Regression beta coefficients of a group dummy variable [subclinical psychotic experience (SPE)-negative = 0, SPE-positive = 1] to laterality indices (LIs) of pallidal volume controlling for the effect of age (Model 1); and no covariates (Model 2) in (a) boys and (b) girls in Tanner stage 1.

(a)

| Beta | SE | Cohen's *d* | SE of Cohen's *d* | *t* value | *p* value |  |
| --- | --- | --- | --- | --- | --- | --- |
| 0.07 | 0.03 | 1.05 | 0.52 | 2.02 | 0.058 | Model 1 |
| 0.06 | 0.03 | 0.94 | 0.48 | 1.96 | 0.064 | Model 2 |

(b)

| Beta | SE | Cohen's *d* | SE of Cohen's *d* | *t* value | *p* value |  |
| --- | --- | --- | --- | --- | --- | --- |
| 0.06 | 0.03 | 0.94 | 0.39 | 2.42 | 0.025 | Model 1 |
| 0.07 | 0.03 | 1.08 | 0.40 | 2.71 | 0.013 | Model 2 |

Abbreviation: SE, standard error.

**Supplementary Table 22** Regression beta coefficients of the Adolescent Psychotic-like Symptom Screener (APSS) total scores to left and right pallidal volume controlling for the effect of age, sex, and intracranial volume (ICV) (Model 1); no covariates (Model 2); age (Model 3); age and sex (Model 4); and age, sex, ICV and Tanner stage (Model 5).

|  | Beta (mm^3^) | SE (mm^3^) | *t* value | *p* value |  |
| --- | --- | --- | --- | --- | --- |
| Lpal | 4.61 | 11.67 | 0.39 | 0.694 | Model 1 |
|  | 13.02 | 12.55 | 1.04 | 0.301 | Model 2 |
|  | 11.41 | 12.52 | 0.91 | 0.363 | Model 3 |
|  | 9.57 | 12.19 | 0.79 | 0.433 | Model 4 |
|  | 6.05 | 11.75 | 0.51 | 0.607 | Model 5 |
| Rpal | -5.78 | 8.98 | -0.64 | 0.521 | Model 1 |
|  | 0.49 | 9.93 | 0.05 | 0.961 | Model 2 |
|  | 0.62 | 9.99 | 0.06 | 0.950 | Model 3 |
|  | -1.16 | 9.58 | -0.12 | 0.904 | Model 4 |
|  | -5.87 | 9.06 | -0.65 | 0.518 | Model 5 |

Abbreviations: SE, standard error; Lpal, left pallidum; Rpal, right pallidum.

**Supplementary Table 23** Regression beta coefficients of the Adolescent Psychotic-like Symptom Screener (APSS) total scores to laterality indices (LIs) of pallidal volume controlling for the effect of age and sex (Model 1); no covariates (Model 2); age (Model 3); and age, sex, and Tanner stage (Model 4).

| Beta | SE | *t* value | *p* value |  |
| --- | --- | --- | --- | --- |
| 0.003 | 0.003 | 0.95 | 0.343 | Model 1 |
| 0.004 | 0.003 | 1.10 | 0.273 | Model 2 |
| 0.003 | 0.003 | 0.95 | 0.344 | Model 3 |
| 0.004 | 0.003 | 1.06 | 0.292 | Model 4 |

Abbreviation: SE, standard error.

**Supplementary Table 24** Regression beta coefficients of the Adolescent Psychotic-like Symptom Screener (APSS) total scores to left and right pallidal volume controlling for the effect of age, sex, and intracranial volume (ICV) (Model 1); no covariates (Model 2); age (Model 3); and age and sex (Model 4) in adolescents in Tanner stage 1.

|  | Beta (mm^3^) | SE (mm^3^) | *t* value | *p* value |  |
| --- | --- | --- | --- | --- | --- |
| Lpal | 50.94 | 24.08 | 2.12 | 0.041 | Model 1 |
|  | 54.61 | 25.41 | 2.15 | 0.037 | Model 2 |
|  | 52.06 | 24.38 | 2.14 | 0.039 | Model 3 |
|  | 56.59 | 24.27 | 2.33 | 0.025 | Model 4 |
| Rpal | -14.92 | 18.29 | -0.82 | 0.419 | Model 1 |
|  | -15.03 | 19.69 | -0.76 | 0.449 | Model 2 |
|  | -15.78 | 19.79 | -0.80 | 0.430 | Model 3 |
|  | -9.54 | 18.74 | -0.51 | 0.613 | Model 4 |

Abbreviations: SE, standard error; Lpal, left pallidum; Rpal, right pallidum.

**Supplementary Table 25** Regression beta coefficients of the Adolescent Psychotic-like Symptom Screener (APSS) total scores to laterality indices (LIs) of pallidal volume controlling for the effect of age and sex (Model 1); no covariates (Model 2); and age (Model 3) in adolescents in Tanner stage 1.

| Beta | SE | *t* value | *p* value |  |
| --- | --- | --- | --- | --- |
| 0.019 | 0.007 | 2.50 | 0.016 | Model 1 |
| 0.020 | 0.007 | 2.67 | 0.011 | Model 2 |
| 0.019 | 0.007 | 2.64 | 0.012 | Model 3 |

Abbreviation: SE, standard error.

**SUPPLEMENTARY FIGURE**

**Supplementary Figure 1** A histogram of 7-item Adolescent Psychotic-like Symptom Screener (APSS) scores in the current study sample.
